# A molecular dynamics simulation study decodes the Zika virus NS5 methyltransferase bound to SAH and RNA analogue

**DOI:** 10.1038/s41598-018-24775-4

**Published:** 2018-04-20

**Authors:** Chih-Hung Chuang, Shean-jaw Chiou, Tian-Lu Cheng, Yeng-Tseng Wang

**Affiliations:** 1 Department of Biochemistry, School of Medicine, College of Medicine, Kaohsiung Medical University, Kaohsiung, Taiwan; 2 Center for Biomarkers and Biotech Drugs, Kaohsiung Medical University, Kaohsiung, Taiwan; 3 Graduate Institute of Medicine, Kaohsiung Medical University, Kaohsiung, Taiwan; 4 Department of Medical Research, Kaohsiung Medical University Hospital, Kaohsiung, Taiwan; 5 Department of Medical Laboratory Science and Biotechnology, Kaohsiung Medical University Hospital, Kaohsiung, Taiwan

## Abstract

Since 2015, widespread Zika virus outbreaks in Central and South America have caused increases in microcephaly cases, and this acute problem requires urgent attention. We employed molecular dynamics and Gaussian accelerated molecular dynamics techniques to investigate the structure of Zika NS5 protein with S-adenosyl-L-homocysteine (SAH) and an RNA analogue, namely 7-methylguanosine 5′-triphosphate (m7GTP). For the binding motif of Zika virus NS5 protein and SAH, we suggest that the four Zika NS5 substructures (residue orders: 101–112, 54–86, 127–136 and 146–161) and the residues (Ser56, Gly81, Arg84, Trp87, Thr104, Gly106, Gly107, His110, Asp146, Ile147, and Gly148) might be responsible for the selectivity of the new Zika virus drugs. For the binding motif of Zika NS5 protein and m7GTP, we suggest that the three Zika NS5 substructures (residue orders: 11–31, 146–161 and 207–218) and the residues (Asn17, Phe24, Lys28, Lys29, Ser150, Arg213, and Ser215) might be responsible for the selectivity of the new Zika virus drugs.

## Introduction

Zika virus belongs to the *Flavivirus* genus and is transmitted by the *Aedes aegypti* and *Aedes albopictus* mosquitoes. Since 2015, widespread Zika virus outbreaks in Central and South America have caused increases in microcephaly cases. Microcephaly is a birth defect that results in babies born with parts of the brain and skull missing^1^. The Zika virus contains a positive single strand RNA genome of approximately 11 kb that encodes a polyprotein, which leads to three structural proteins, namely the envelope (E), membrane precursor (PrM), and capsid (C), and seven nonstructural proteins (NS1, NS2A, NS2B, NS3, NS4A, NS4B, and NS5)^2^. The three structural proteins contribute to the viral particles and the seven nonstructural proteins contribute to viral replication. The E protein is a major component of the viral surface and is involved with aspects of viral replication such as membrane fusion and host cell binding^2^. The NS1, NS3, and NS5 nonstructural proteins are large and highly-conserved proteins, whereas the NS2A, NS2B, NS4A, and NS4B nonstructural proteins are small and hydrophobic^3^. Grant *et al*. showed that NS5 is a promising target for vaccines and drugs against Zika and related viruses^4^. The 103-kDa NS5 protein is the largest viral protein whose C-terminal portion has RNA-dependent RNA polymerase activity and N-terminal RNA cap-processing activity (methyltransferase domain)^5,6^. In addition, Zika NS5 can promote the proteasomal degradation of host signal transducer and can activate transcription 2 (STAT2) to inhibit type I interferon (IFN) signalling and thus antagonize the host antiviral response^4^. The full-length Zika NS5 proteins were resolved by Li *et al*. (Protein Data Bank (PDB) ID: 5u0b; NS5 bound to S-adenosyl-L-homocysteine (SAH))^7^. The x-ray structure is the initial target used in our binding mechanism studies.

Computational simulations have been applied to study Zika virus-related proteins^8^. Unfortunately, these modelling structures contain insufficient inhibitor-protein interaction information. Understanding the conformational dynamics of inhibitor-protein structures, and the energy produced by inhibitor-protein interactions is crucial for translating conformational samplings into functional efficacies. The process of inhibitor-protein binding occurs in a microsecond, making detailed conformational states difficult to achieve with experimental data. Currently, two major issues occur due to (1) the conformational states of protein receptors between binding and unbinding states and (2) the pathway of the inhibitor-protein interaction process.

All-atoms molecular dynamics (MD) simulations remain limited to the conformational ensembles obtained from a single long-time-scale conventional molecular dynamics (cMD) simulation due to the possible energy barriers between various intermediate states. Therefore, a multiscale simulation method that combines an enhanced sampling technique, which can take samples at various intermediate states, with an all-atoms simulation is required. Enhanced sampling techniques have been successfully applied to calculate the free-energy profiles^9^ and to perform conformational sampling through accelerated molecular dynamics (aMD)^10^. These enhanced sampling methods can provide key insights into the free-energy profiles and intermediate protein structures. In general, the disadvantage of enhanced sampling techniques is the requirement to predefine protein structures, potential energies, additional energies, and reaction coordinates. Simulations using aMD or Gaussian aMD (GaMD) are enhanced sampling methods that can avoid such requirements. Through aMD, a boost of potential energy is added to the potential energy surface system resulting in a decreased energy barrier, which enables the acceleration of the transitions between low-energy states^10–12^. This method has been successfully applied to simulations of biological systems, and hundreds-of-nanoseconds aMD simulations can yield the same results as millisecond cMD simulations^13–16^.

In recent studies, the aMD method has produced substantial energetic noise during reweighting^17^. In aMD simulations, the applied boost potential is typically of the order of tens-to-hundreds of kilocalories per mole, which is usually greater than those of other enhanced sampling methods. Accurately reweighting aMD simulations has been problematic, especially for large protein molecules^18^. By introducing GaMD, Miao *et al*. provided an approach to improving the aMD method. The boost potential of the GaMD method follows a near-Gaussian distribution, for which the cumulated second-order expansion improves the reweighting of aMD simulations^19^. The reweighted free-energy profiles yielded by GaMD are in close agreement with long-time-scale cMD simulations^20^.

In the present study, the experimental biological binding affinities^21^ (IC50) of SAH and 7-methylguanosine 5′-triphosphate (m7GTP) were compared using GaMD simulations; these compounds are listed in Table 1. The transfer function (ΔGbind = −*RT* ln(IC50)) is used to convert the IC50 values into experimental ΔGbind values, which are listed in Table 1. We applied GaMD to simulate the Zika virus NS5 protein with SAH and m7GTP. Starting from the full-length of the Zika NS5 x-ray structure, the interaction of the two molecules with the Zika virus NS5 protein was studied. We demonstrated that GaMD simulation enables a detailed analysis of the interaction of these two molecules with the Zika NS5 protein.

**Table 1:** Experimental data of the Zika NS5 protein with SAH and RNA analogues^21^.

Name	IC50 (μM)	ΔGbind(kcal/mol) (experimental)	ΔGbind(kcal/mol) (Our GaMD)	ΔGbind(kcal/mol) (WHAM/umbrella sampling)
SAH	0.43	8.82	8.42 ± 1.98	7.54
M7GTP	184.00	5.17	5.21 ± 1.21	5.91

^*^The 2D structures are shown in Fig. S25.

## Theoretical Calculations Methods

### Gaussian accelerated molecular dynamics

GaMD is an enhanced conformational sampling method for biomolecules that adds a harmonic boost potential to smooth the system potential energy surface^19^. When the system potential (*V*) is lower than a referenced energy (*E*), a harmonic boost potential (Δ*V*) is added as follows:

1 $${\rm{\Delta }}V=\frac{1}{2}K{(E-V)}^{2},\,if\,V < E$$

where *K* is a harmonic force constant. The modified system potential (V*) is given by

2 $${V}^{\ast }=V+\frac{1}{2}K{(E-V)}^{2},if\,V < E\,$$

If the system potential (*V*) is greater than a referenced energy (*E*), the harmonic boost potential (Δ*V*) is equal to zero. By smoothing the potential energy surface for overcoming intermedia energy barriers, the boost potential satisfies the following step. For two potential energy values *V*1 and *V*2, assume that *V*1 < *V*2 and the biased *V*1* < *V*2*. By replacing V* with equation (2), the relationship is expressed as follows:

3 $$E < \frac{1}{2}(V1+V2)+\frac{1}{K}$$

Step (1) If *V*1 < *V*2, the potential difference on the smoothed energy surface should be smaller than that of the original energy surface. By replacing V* with equation (2), the relationship is expressed as

4 $$E > \frac{1}{2}(V1+V2)$$

Step (2) Combing equations (3) and (4), and the relationship *Vmin* ≤ *V*1 < *V*2 ≤ *Vmax*, we can derive

5 $$Vmax\le E\le Vmin+\frac{1}{K}$$

where *Vmin* and *Vmax* are the minimum and maximum potential energies.

Step (3) By equation (5), we can obtain

6 $$\frac{1}{K}\le \frac{1}{Vmax-Vmin}$$

where *K* constant is defined as

7 $$K=K0(\frac{1}{Vmax-Vmin}),0 < K0\le 1$$

and *K*0 is the magnitude of the applied boost potential.

Step (4) The standard deviation (SD) of Δ*V* must be sufficiently small to ensure accurate reweighting^22^.

8 $${\sigma }_{{\rm{\Delta }}V}=\sqrt{{(\frac{\partial {\rm{\Delta }}V}{\partial V}|V=Vave)}^{2}{{\sigma }_{V}}^{2}}=K(E-Vave){\sigma }_{V}\le {\sigma }_{0}$$

where the *Vave* and *σ*_*V*_ are the average and SD of the potential energies, and *σ*_Δ*V*_ is the SD of Δ*V* with *σ*0 as a user-specified upper limit for accurate reweighting of potential energies. In our simulations, the SDs of the total potential and dihedral potential boosts are 10 kcal/mol.

Step (5) To extend step (2), if *E* = *Vmax*, we can use equation (5) to obtain

9 $$K0\le \frac{\sigma 0}{\sigma V}\frac{Vmax-Vmin}{Vmax-Vave}$$

According to equations (21) and (19), *K*0 can be defined as:

10 $$K0=min\{1.0,\frac{\sigma 0}{\sigma V}\frac{Vmax-Vmin}{Vmax-Vave}\}$$

Step (6) To extend step (2), if *E* = *Vmin* + 1/*k*, we can use equation (8) to obtain

11 $$K0\ge (1-\frac{\sigma 0}{\sigma V})\frac{Vmax-Vmin}{Vmax-Vave}$$

Step (7) GaMD provides the total potential boost, dihedral potential boost, and dual potential boost in order to accelerate the molecular simulations. The boost potential (Δ*V*) is given as follows:

12 $${\rm{\Delta }}V=\frac{1}{2}K0\frac{1}{{V}_{max}-{V}_{min}}{(E-V)}^{2},if\,V < E$$

where *K*0 is the magnitude of the applied boost potential, and *Vmin* and *Vmax* are the minimum and maximum potential energies of the system. Initially, *K*0 is equal to 1.0, and *Vmax* and *Vmin* are obtained through cMD simulations. The distribution and anharmonicity of the GaMD method were applied to the alanine dipeptide, chignolin, and lysozyme simulations to characterize the extent to which Δ*V* follows a Gaussian distribution^19^.

### Reweighted free-energy calculations for GaMD simulations

The probability distribution of the selected reaction^22^ coordinate *A*(*r*) is defined as P*(*A*), where *r* can be distance, angle, root-mean-square deviation, or other factors. Using the GaMD boost energies of each reaction coordinate, P*(*A*) can be reweighted and defined as

13 $$P({A}_{j})={P}^{\ast }({A}_{j})\frac{{\langle {e}^{\beta {\rm{\Delta }}V(r)}\rangle }_{j}}{{\sum }_{j=1}^{M}{\langle {e}^{\beta {\rm{\Delta }}V(r)}\rangle }_{j}},J=1 \sim M$$

where *M* is the number of bins, *β* is equal to the KBT, and ${\langle {e}^{\beta \Delta V(r)}\rangle }_{j}$ is the ensemble-average factor of the *j*th bin. For reducing the energetic noise, the ensemble-average factor can be defined as follows:

14 $$\langle {e}^{\beta {\rm{\Delta }}V(r)}\rangle =exp\{\sum _{K=1}^{\infty }\frac{{\beta }^{K}}{K!}{C}_{K}\}$$

According to equation (14), the first three cumulants can be defined as follows:

15 $$\begin{array}{c}C1={\rm{\Delta }}V,\\ C2={\rm{\Delta }}{V}^{2}-\,{\rm{\Delta }}{V}^{2},\\ C3={\rm{\Delta }}{V}^{3}-3{\rm{\Delta }}{V}^{2}{\rm{\Delta }}V+2{\rm{\Delta }}{V}^{2}\end{array}$$

The reweighted free energies can then be calculated by

16 $$F({A}_{j})=-\frac{1}{\beta }lnP({A}_{j})$$

### Free-energy calculations (WHAM) for umbrella sampling simulations

A harmonic potential was applied to the stretching^23^ constraints, i.e. the distance constraints between the center of mass of the ligands and the binding pockets (Figs S1 and S7) with a force constant of 10.0 kcal/mol. The RC1 reaction coordinates follow the distance from the centre of the SAH mass to the centre of mass defined by the residues that shape the binding pocket (Gly86, Trp87, Thr104, Lys105, Asp131, Val132, Asp146, and Ile147; Fig. S1). The RC2 reaction coordinates follow the distance from the centre of the m7GTP mass to the centre of mass defined by the residues that shape the binding pocket (Lys13, Leu16, Asn17, Met19, Ser150, Ser151, and Ser215; Fig. S7). The RC1 value varied from 3.0 to 24.0 Å in 1 Å increments. The RC2 value varied from 6.0 to 22.0 Å in 1 Å increments. The MD simulations for PMF determination were performed with an initial 5-ns equilibration followed by 10-ns sampling at a given reaction coordinate value. Moreover, the umbrella sampling simulations were performed with GENESIS 1.2.0 software^24^. Then the free energy profiles (PMF) were analysed with the WHAM software^25^.

### GaMD simulation of the Zika virus NS5 protein system

First, we modified a partial length of the Zika NS5 protein structure (PDB ID: 5kqs), and we used PyMOL software to modify the ligand (m7GDP to m7GTP). Second, we aligned the partial length of the Zika NS5 protein structure and the full length of the Zika NS5 protein structure (PDB ID: 5u0b). We then inserted the ligand (m7GTP) into the full-length Zika NS5 protein structure. The initial complex structures are Zika NS5 with SAH and Zika NS5 with m7GTP. These complex structures were generated and then inserted into TIP3P solvent molecules. The size of the complex structures was approximately 10.00 × 11.00 × 11.00 nm^3^. These initial complexes were then simulated using the AMBER 16 package with the AMBER FF12SB all-hydrogen amino acid and amber GAFF parameters. The geometries of the SAH and m7GTP were fully optimized, and their electrostatic potentials were obtained using a single-point calculation at the Hatree–Fock level with the 6–31 G(d,p) basis set using the GAUSSIAN 09 program^26^. Subsequently, partial charges were obtained by employing the restrained electrostatic potential procedure using the Antechamber package. All cMD simulations were performed in the isothermal–isobaric (NPT) assembly with a simulation temperature of 310 K, unless stated otherwise, by using the Verlet integrator with an integration time step of 0.002 ps and SHAKE constraints^27^ for all covalent bonds involving hydrogen atoms. In the electrostatic interactions, atom-based truncation was performed using the PME^28^ method, and the switch van der Waals function was used with a 2.00 nm cut-off for atom-pair lists. These complex structures were minimized for 100,000 conjugate gradient steps and then subjected to a 100-ns, isothermal, constant-volume MD simulation. Moreover, the final structures from these simulations were used in five dependent 5000-ns GaMD simulation calculations, and these structures were used in the umbrella sampling simulations.

### Reweighted Free-energy calculation through potential of mean force and preresidue displacement

For the Zika NS5 protein with SAH (RC1 = 3–8 Å) and m7GTP (RC2 = 6–11 Å), all residues were obtained using preresidue displacement calculations. The preresidue displacement calculations were applied to the major barriers: RC1 = 3–8 Å and RC2 = 6–11 Å). The reaction coordinate profiles and preresidue displacement calculations were analysed using the AmberTools 16 and VMD software packages. The reaction coordinates profiles were calculated for the reaction coordinates used for the potential of mean force (PMF) calculations. The PyReweighting toolkit^22^ was used to reweight the GaMD simulations for the PMF profile calculations and to examine the boost potential distributions. One-dimensional PMF profiles were also constructed using the reaction coordinates for the Zika NS5 protein with a bin size of 1.0 Å. When the number of simulation frames within a bin was lower than a certain limit, the bin was insufficiently sampled and thus was excluded for reweighting.

## Results and Discussion

### Potential of mean force calculation for Zika virus NS5 proteins: SAH- and m7GTP-complex GaMD simulations

**Figure 1:**
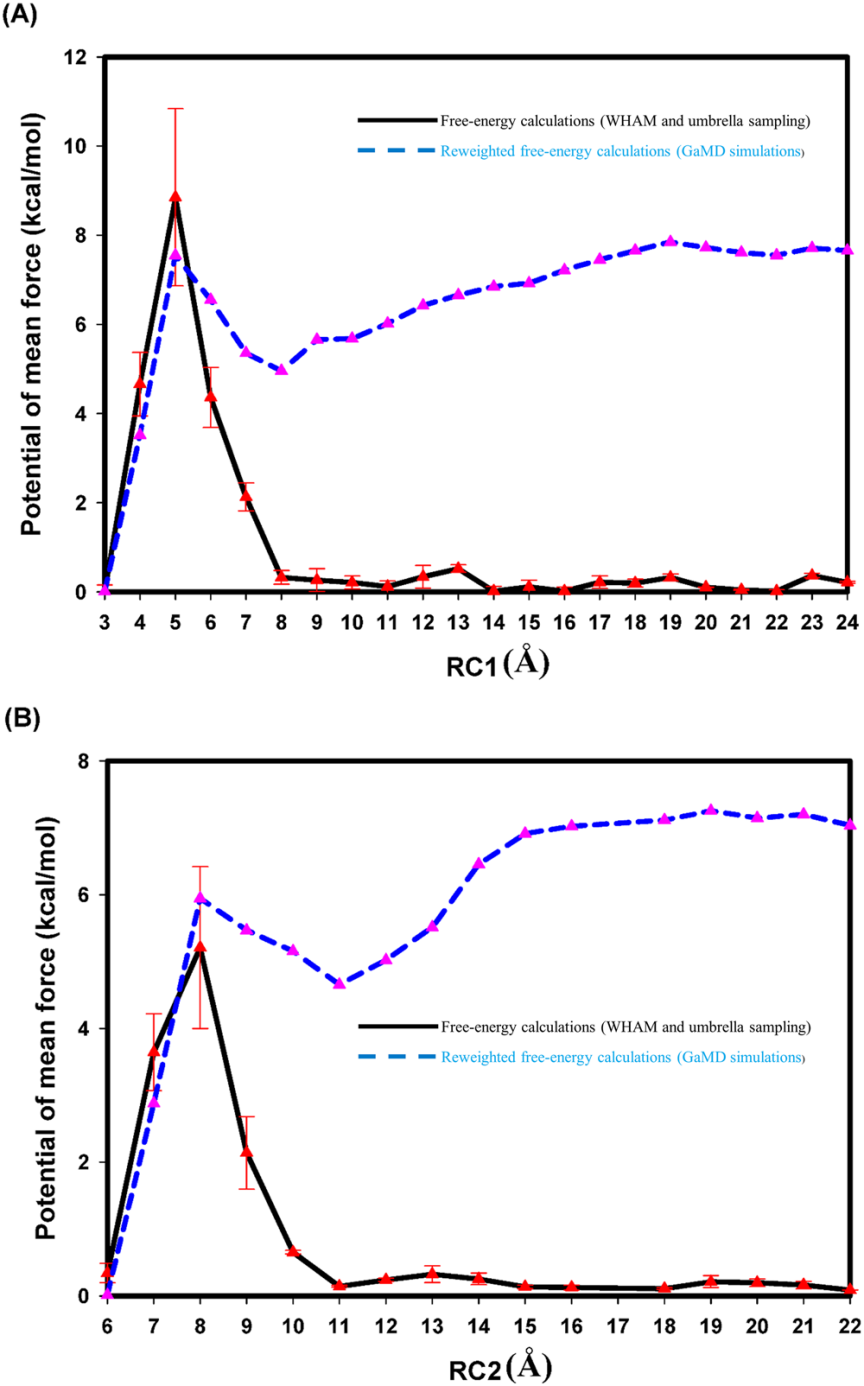
Free-energy profiles (PMF) at the reaction coordinates. The PMF profiles were calculated using 5000-ns GaMD simulations and umbrella sampling simulations. (**A**) Zika NS5 protein with SAH. (**B**) Zika NS5 protein with m7GTP.

Following the original paper “Reweighted free-energy calculations (PyReweighting-1D.py),”^22^ we used five individual and independent 5000-ns GaMD simulations to estimate the error bar and average of the PMF, a measure of free energy, using the second-order cumulant expansion method^22^. Our results are listed in Table 1 and Fig. 1. Miao *et al.*^22^. indicated that energy barriers were underestimated in the PyReweighting-1D.py program, leading to obvious fluctuations in the reweighted free-energy calculations. Our PMF calculations conformed to the results^22^. For the Zika virus NS5 protein and SAH complex, there is a major energy barrier of 8.42 ± 1.98 kcal/mol at RC1 = 3–8 Å. When RC1 = 5 Å, the complex reaches the top of the energy barrier. For the Zika virus NS5 protein and m7GTP complex, there is a major energy barrier of 5.21 ± 1.21 kcal/mol at RC2 = 6–11 Å. When the RC2 = 8 Å, the complex reaches the top of the energy barrier. Our energy barrier predictions were relatively close to the experimental data.

### Potential of mean force calculation for Zika virus NS5 proteins: SAH- and m7GTP-complex umbrella sampling simulations

Our results are listed in Fig. 1. For the Zika virus NS5 protein and SAH complex, there is a major energy barrier of 7.54 kcal/mol at RC1 = 3–8 Å. When RC1 = 5 Å, the complex reaches the top of the energy barrier. For the Zika virus NS5 protein and m7GTP complex, there is a major energy barrier of 5.91 kcal/mol at RC2 = 6–11 Å. When the RC2 = 8 Å, the complex reaches the top of the energy barrier. Our energy barrier predictions were relatively close to our reweighted free-energy calculations.

### Functionally important residues and preresidue displacement

**Figure 2:**
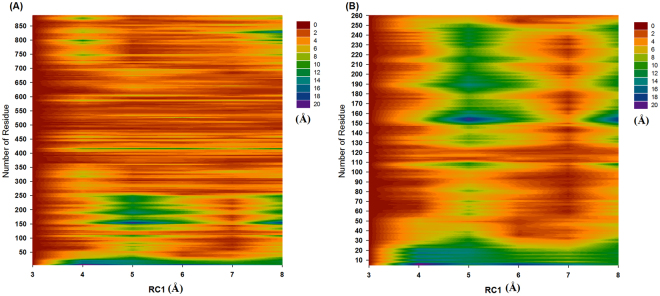
Analysis of the preresidue displacement of SAH. (**A**) Full length of the Zika NS5 protein and (**B**) partial length of the Zika NS5 protein (residue orders: 5–260).

**Figure 3:**
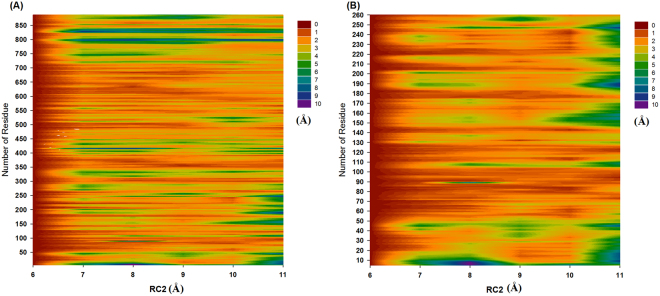
Analysis of the preresidue displacement of m7GTP. (**A**) Full length of the Zika NS5 protein and (**B**) partial length of the Zika NS5 protein (residue orders: 5–260).

The identification of functionally important residues can provide clear insight into the structural aspects of the Zika NS5 proteins. In this work, the structure-based approach was applied to identify the functionally important residues of the major energy barriers at RC1 = 3–8 and RC2 = 6–11 Å. From the complex structures at RC1 = 3–8 and RC2 = 6–11 Å, the important residues and pharmacophore regions were analysed using the Ligandscout program. Because there were many snapshots, residues with probability of >0.5 were selected for the binding mode analysis. Our results are listed in Tables 2 and 3 (Figs S1–S12 present the representative snapshots at RC1 = 3–8 and RC2 = 6–11 Å). The preresidue displacement calculations in Figs 2 and 3 show that the partial length of the Zika NS5 protein (residue: 5–260) can affect the binding ability of the two analogues. For the preresidue displacement analysis of the SAH, the four Zika NS5 substructures (residue orders: 101–112, 54–86, 127–136, and 146–161) had clear fluctuations; the results are presented in Table 4 and Figs S13–S17. For the preresidue displacement analysis of m7GTP, the three Zika NS5 substructures (residue orders: 11–31, 146–161, and 207–218) had clear fluctuations; the results are displayed in Table 5 and Figs S18–S22.

**Table 2:** Analysis of the Zika virus NS5 protein and SAH complex binding modes at RC1 = 3–8 Å.

RC1 (Å)	Elestostatic	Van der Waals	Hydrogen bonding
3	Trp87 and Ile147	Trp104, Asp146 and ILE147	Gly86, Lys105, Asp131 and Val132
4	Arg84 and Asp146	ILE147	Ser56, Arg84, Thr104, Gly106, Gly107 and Asp146
5	Arg84 and Asp146	ILE147	Ser56, Arg84, Thr104, Asp146 and Gly148
6	Arg84, Trp87 and Asp146	ILE147	Ser56, Arg84, Asp146 and Gly148
7	Arg84 and Asp146	ILE147	Ser56, Arg84, His110 and Asp146
8	Asp146, Lys182 and Arg213		Gly81, Asp146, Ser150 and Arg213

**Table 3:** Analysis of the Zika virus NS5 protein and m7GTP complex binding modes at RC2 = 6–11 Å.

RC2 (Å)	Elestostatic	Van der Waals	Hydrogen bonding
6			Lys13, Leu16, Asn17, Met19, Ser150, Ser151 and Ser215
7	Lys28 and Arg213		Asn17, Lys28, Ser150, Arg213 and Ser215
8	Lys28 and Arg213		Asn17, Lys28, Arg213 and Ser215
9	Phe24, Lys28 and Arg213		Lys28, Arg213 and Ser215
10	Lys28 and Arg213		Lys28, Arg213 and Ser215
11	Lys28, Lys29 and Arg213		Lys28 and Arg213

**Table 4:** Analysis of the Zika virus NS5 protein and SAH complex preresidue displacements at RC1 = 3–8 Å.

Range RC1 (Å)	residues
3–4	Residue:101–112, 127–136 and 146–161
4–5	Residue:101–112, 54–86, 127–136 and 146–161
5–6	Residue:101–112, 54–86, 127–136 and 146–161
6–7	Residue: 146–161
7–8	Residue:101–112, 54–86, 127–136 and 146–161

**Table 5:** Analysis of the Zika virus NS5 protein and m7GTP complex preresidue displacements at RC2 = 6–11 Å.

Range RC2 (Å)	residues
6–7	Residue:11–31, 146–161 and 207–218
7–8	
8–9	Residue:11–31, 146–161 and 207–218
9–10	Residue:11–31, 146–161 and 207–218
10–11	Residue:11–31, 146–161 and 207–218

### Decoding the binding of SAH with Zika virus NS5 proteins

Our PMF profiles revealed that SAH must overcome a major energy barrier of 8.42 kcal/mol at RC1 = 3–8 Å; then, this molecule can bind with the binding pockets of the Zika NS5 protein (Gly86, Trp87, Thr104, Lys105, Asp131, Val132, Asp146, and Ile147). In addition SAH must overcome the four Zika NS5 substructures (residue orders: 101–112, 54–86, 127–136, and 146–161). At RC1 = 8 Å, SAH interacts with the Gly81, Asp146, Ser150 Lys182, and Arg213 residues, causing fluctuations in the four Zika NS5 substructures (residue orders: 101–112, 54–86, 127–136, and 146–161).

At RC1 = 7 Å, SAH interacts with the Ser56, Arg84, His110, Asp146, and ILE147 residues, causing fluctuations in one Zika NS5 substructure (residue orders: 146–161). At RC1 = 6 Å, SAH interacts with the Ser56, Arg84, Trp87, Asp146, ILE147, and Gly148 residues, causing fluctuations in the four Zika NS5 substructures (residue orders: 101–112, 54–86, 127–136, and 146–161). At RC1 = 5 Å, SAH interacts with the Ser56, Arg84, Thr104, Asp146, ILE147, and Gly148 residues, causing fluctuations in the four Zika NS5 substructures (residue orders: 101–112, 54–86, 127–136, and 146–161). At RC1 = 4 Å, SAH interacts with the Ser56, Arg84, Thr104, Gly106, Gly107, Asp146, and ILE147 residues, causing fluctuations in three Zika NS5 substructures (residue orders: 101–112, 127–136, and 146–161). Our predicted binding mechanisms indicated that the four Zika NS5 substructures (residue orders: 101–112, 54–86, 127–136, and 146–161) and the Ser56, Gly81, Arg84, Trp87, Thr104, Gly106, Gly107, His110, Asp146, Ile147, and Gly148 residues affect the ability of the full-length Zika NS5 protein to bind with SAH.

### Decoding the binding of m7GTP with Zika virus NS5 proteins

Our PMF profiles revealed that m7GTP must overcome a major energy barrier of 5.21 kcal/mol at RC2 = 6–11 Å; then, this molecule can bind with the binding pockets of the Zika NS5 protein (Lys13, Leu16, Asn17, Met19, Ser150, Ser151, and Ser2152). In addition, m7GTP must overcome three Zika NS5 substructures (residue orders: 11–31, 146–161, and 207–218). At RC2 = 11 Å, m7GTP interacts with the Lys28, Lys29, and Arg213 residues, causing fluctuations in the three Zika NS5 substructures (residue orders: 11–31, 146–161, and 207–218). At RC2 = 10 Å, m7GTP interacts with the Lys28, Arg213, and Ser215 residues, causing fluctuations in the three Zika NS5 substructures (residue orders: 11–31, 146–161, and 207–218). At RC2 = 9 Å, m7GTP interacts with the Phe24, Lys28, Arg213, and Ser215 residues, causing fluctuations in the three Zika NS5 substructures (residue orders: 11–31, 146–161, and 207–218). At RC2 = 8 Å, m7GTP interacts with the Asn17, Lys28, Arg213, and Ser215 residues, causing no Zika NS5 substructure fluctuations. At RC2 = 7 Å, m7GTP interacts with the Asn17, Lys28, Ser150, Arg213, and Ser215 residues, causing fluctuations in the three Zika NS5 substructures (residue orders: 11–31, 146–161, and 207–218). Our predicted binding mechanisms indicated that the three Zika NS5 substructures (residue orders: 11–31, 146–161, and 207–218) and the Asn17, Phe24, Lys28, Lys29, Ser150, Arg213, and Ser215 residues affect the ability of the full-length Zika NS5 protein to bind with m7GTP.

### Comparing the SAH binding residues of the Zika virus NS5 proteins with those of other *Flavivirus* NS5 proteins

The Ser56, Gly81, Arg84, Trp87, Thr104, Gly106, Gly107, His110, Asp146, Ile147, and Gly148 residues (PDB ID: 5U0B) were selected for comparison with other Zika NS5 proteins (PDB IDs: 5GOZ, 5GP1, 5KQR, 5KQS, 5M5B, 5TFR, 5TMH, 5ULP, 5VIM, 5WXB, 5WZ1, and 5WZ2). Our results indicated that these residues were conserved among the Zika NS5 proteins, and the results are shown in Fig. S23. The Ser56, Gly81, Arg84, Trp87, Thr104, Gly106, Gly107, His110, Asp146, Ile147, and Gly148 residues (PDB ID: 5U0B) were selected for comparison with the other *Flavivirus* NS5 proteins (PDB IDs: 3EVF (Yellow fever), 4V0R (Dengue fever), 2HKS (West Nile fever) and 4K6M (Japanese encephalitis)). Our results indicated that these residues were conserved among the NS5 proteins, and the results are shown in Fig. S24.

### Comparing the m7GTP binding residues of the Zika virus NS5 proteins with those of other *Flavivirus* NS5 proteins

The Asn17, Phe24, Lys28, Lys29, Ser150, Arg213, and Ser215 residues (PDB ID: 5U0B) were selected for comparison with other Zika NS5 proteins (PDB IDs: 5GOZ, 5GP1, 5KQR, 5KQS, 5M5B, 5TFR, 5TMH, 5ULP, 5VIM, 5WXB, 5WZ1, and 5WZ2). Our results indicated that these residues were conserved among the Zika NS5 proteins, and the results are shown in Fig. S23. The Asn17, Phe24, Lys28, Lys29, Ser150, Arg213, and Ser215 residues (PDB ID: 5U0B) were selected for comparison with the other *Flavivirus* NS5 proteins (PDB IDs: 3EVF (Yellow fever), 4V0R (Dengue fever), 2HKS (West Nile fever) and 4K6M (Japanese encephalitis)). The results are shown in Fig. S24. Except for two residues (Lys28 and Lys29), the residues were conserved among the NS5 proteins. The two residues of the West Nile fever, Japanese encephalitis, and Yellow fever viruses were Arg-Lys, Arg-Arg, and Lys-Arg, respectively. The Arg and Lys residues have similar structures and isoelectric points. Thus, we think that these residue differences might have a reduced impact on the binding affinity of NS65 proteins with m7GTP.

## Conclusions

In this article, we used full-length Zika NS5 proteins (PDB ID: 5u0b; NS5 bound to SAH and m7GTP) as our initial structures. We employed 100-ns cMD simulations to optimize the two Zika virus NS5 protein complex structures. The RC1 reaction coordinates were defined as the distance between the centre of mass of SAH and the centre of mass of the binding pocket (Gly86, Trp87, Thr104, Lys105, Asp131, Val132, Asp146, and Ile147; Fig. S1). The RC2 reaction coordinates were defined as the distance between the centre of mass of m7GTP and the centre of mass of the binding pocket (Lys13, Leu16, Asn17, Met19, Ser150, Ser151, and Ser215). Then, we performed GaMD, preresidue displacements, and PMF calculations to predict the binding mechanisms of these molecules with Zika virus NS5 proteins. For the Zika virus NS5 protein and SAH complex, there is a major energy barrier of 8.42 ± 1.98 kcal/mol RC1 = 3–8 Å. For the Zika virus NS5 protein and m7GTP complex, there is a major energy barrier of 5.21 ± 1.21 kcal/mol at RC2 = 6–11 Å. Our energy barrier predictions were similar to the experimental data (Table 1). Moreover, we used the WHAM/umbrella sampling methods to check our reweighted free energy calculations. The energy barrier predictions were relatively close to our reweighted free-energy calculations. For the Zika NS5 protein and SAH complex, our results indicated that the four Zika NS5 substructures (residue orders: 101–112, 54–86, 127–136, and 146–161) and the Ser56, Gly81, Arg84, Trp87, Thr104, Gly106, Gly107, His110, Asp146, Ile147, and Gly148 residues affect the ability SAH to bind with the full-length Zika NS5 protein. For the Zika NS5 protein and m7GTP complex, our results indicated that three Zika NS5 substructures (residue orders: 11–31, 146–161, and 207–218) and the Asn17, Phe24, Lys28, Lys29, Ser150, Arg213, and Ser215 residues influenced Zika NS5 and m7GTP binding through the entrapment of this RNA analogue. Our results also indicated that the two molecules have different binding processes. For the Zika virus NS5 protein and SAH binding motif, our results suggested that the four Zika NS5 substructures (residue orders: 101–112, 54–86, 127–136, and 146–161) and the Ser56, Gly81, Arg84, Trp87, Thr104, Gly106, Gly107, His110, Asp146, Ile147, and Gly148 residues might be responsible for the selectivity of the new Zika virus drug, whereas for the Zika NS5 protein and m7GTP binding motif, our results suggested that three Zika NS5 substructures (residue orders: 11–31, 146–161, and 207–218) and the Asn17, Phe24, Lys28, Lys29, Ser150, Arg213, and Ser215 residues were responsible for the drug selectivity. We also compared our predicted binding residues for SAH and m7GTP with Zika virus with those of other *Flavivirus* NS5 proteins. The results revealed that these residues were conserved among the majority of NS5 proteins.

## Electronic supplementary material

SUPPLEMENTARY INFO
