# Supplementary material for: A molecular dynamics simulation study decodes the Zika virus NS5 methyltransferase bound to SAH and RNA analogue
Source: Sci Rep. 2018 Apr 20;8:6336. doi: 10.1038/s41598-018-24775-4 (PMC5910437; doi:10.1038/s41598-018-24775-4)
Supplement: Supplementary file 1 — SUPPLEMENTARY INFO [file 41598_2018_24775_MOESM1_ESM.doc]

A molecular dynamics simulation study decodes the Zika virus NS5 methyltransferase bound to SAH and RNA analogue

Chih-Hung Chuang^e^ , Shean-jaw Chiou^a^ , Tian-Lu Cheng^ce^ and Yeng-Tseng Wang^abcde*^

^a^.Department of Biochemistry, College of Medicine, Kaohsiung Medical University, Taiwan

^b.^Center for Biomarkers and Biotech Drugs, Kaohsiung Medical University, Kaohsiung, Taiwan

^c.^Graduate Institute of Medicine, Kaohsiung Medical University, Kaohsiung, Taiwan

^d^Department of Medical Research, Kaohsiung Medical University Hospital, Kaohsiung, Taiwan

^e^Department of Medical Laboratory Science and Biotechnology, Kaohsiung Medical University Hospital, Kaohsiung, Taiwan

Corresponding Author: Yeng-Tseng Wang; Fax: +886-07-3218309; Tel: +886-07-3121101; E-mail address: [c00jsw00@kmu.edu.tw](mailto:c00jsw00@kmu.edu.tw); Address: 100,Shih-Chuan 1st Road,Kaohsiung,80708,Taiwan R.O.C.

**
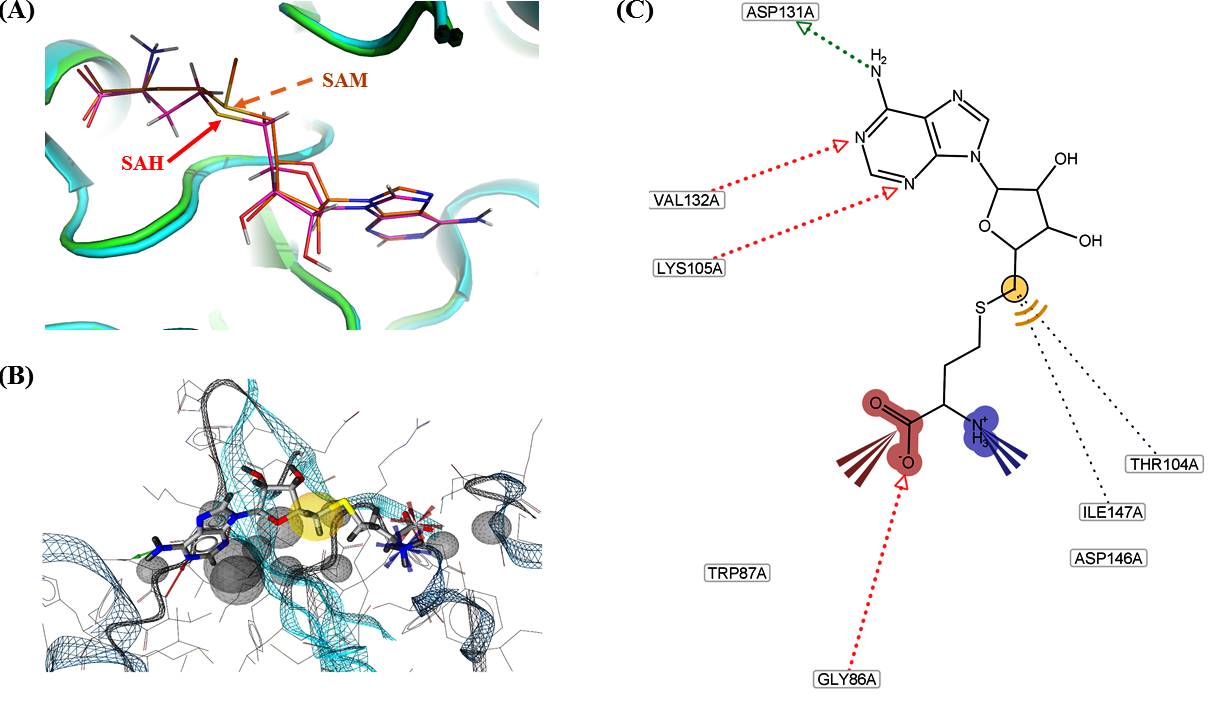
**

**Figure S1.** The experimental binding mode of the full-length of zika NS5 (RC1 at 3 Å), which interact with SAH: (A) Structural alignment of the zika NS5 (PDB ID: 5kqs & colored in cyan) with SAM and the zika NS5 (PDB ID: 5U0b & colored in green) with SAH; (B) the 3D binding modes of the zika NS5 with SAH ; (C) the 2D binding modes of the zika NS5 with SAH

**(A)**

**
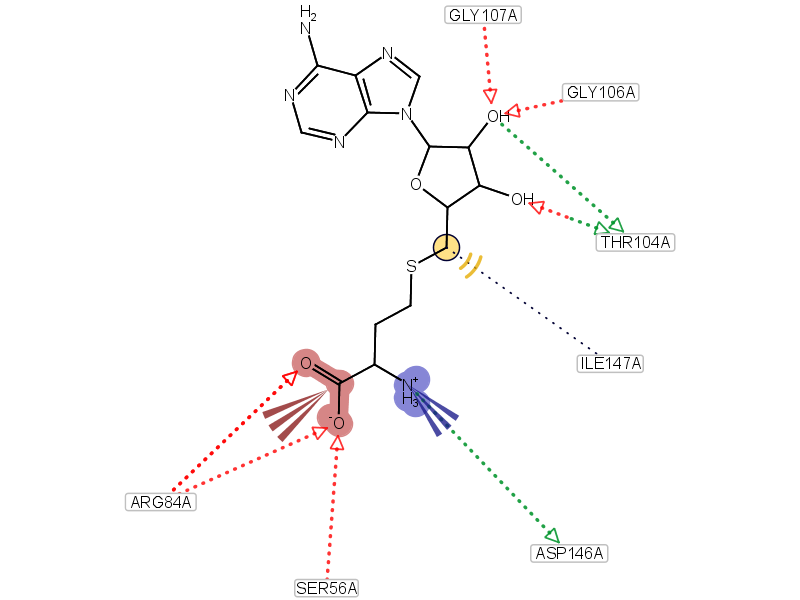
**

**(B)**

**
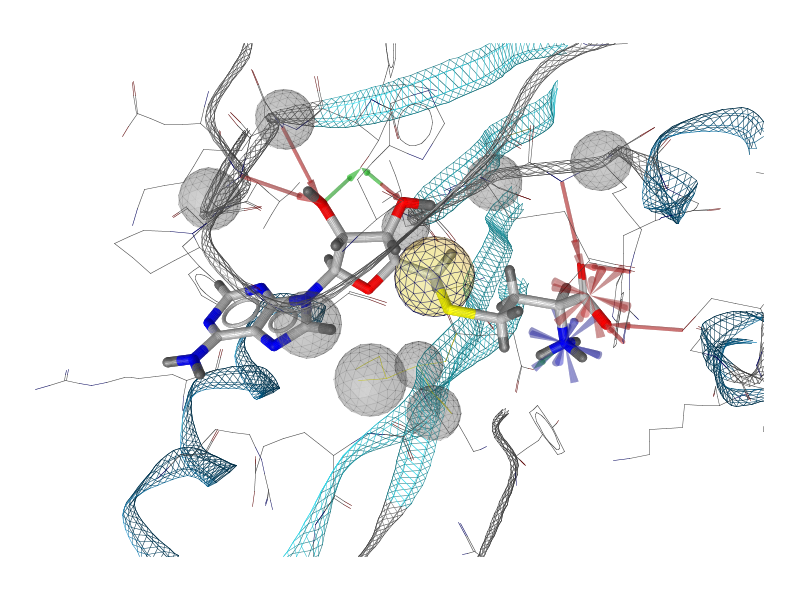
**

**Figure S2.** The predicting binding mode of the full-length of zika NS5 (RC1 at 4 Å), which interact with SAH: (A) 2 D model and (B) 3D mode.

(A)


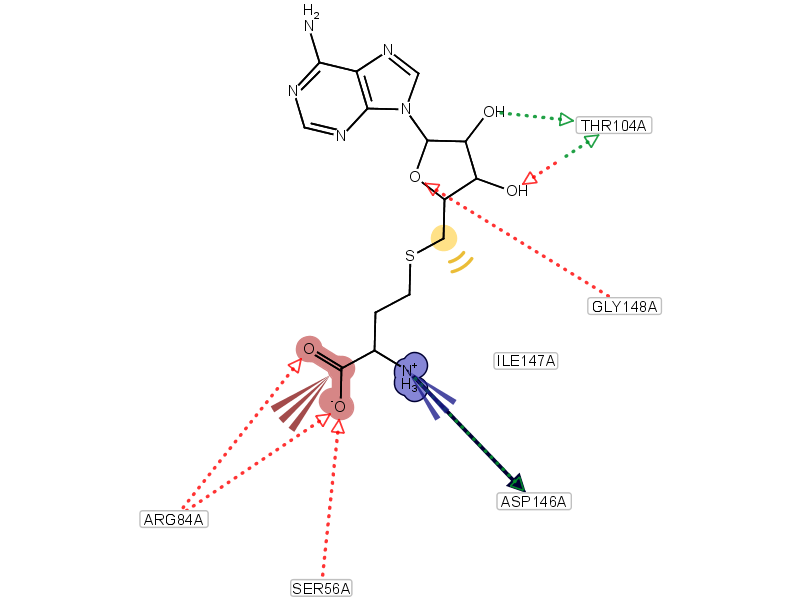


(B)


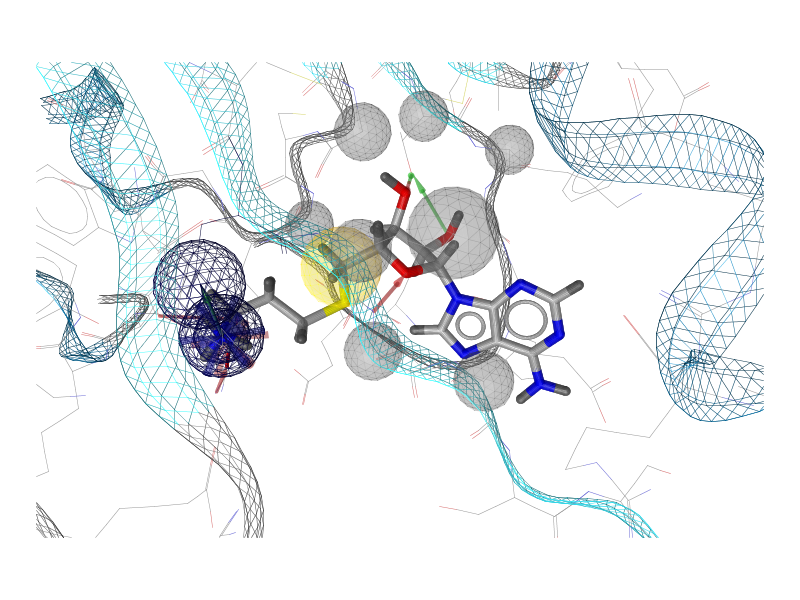


**Figure S3.** The predicting binding mode of the full-length of zika NS5 (RC1 at 5 Å), which interact with SAH: (A) 2 D model and (B) 3D model.

**(A)**

**
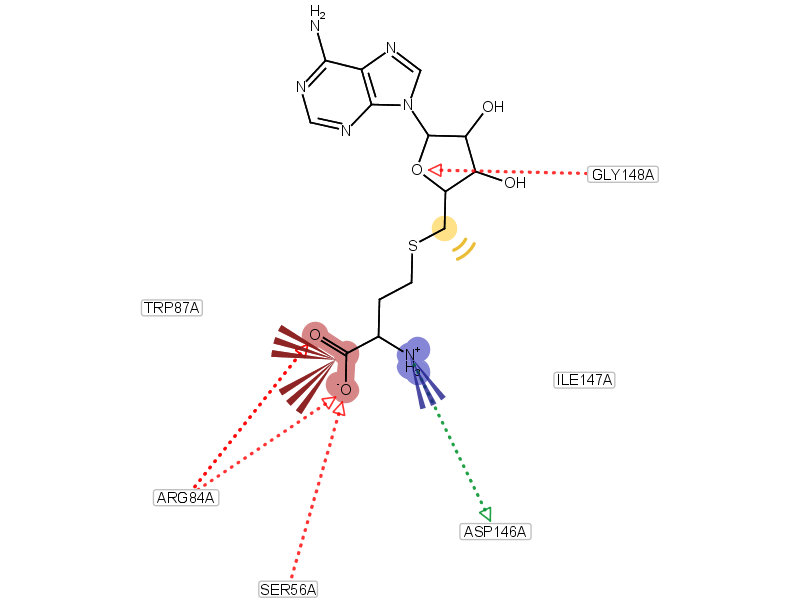
**

**(B)**

**
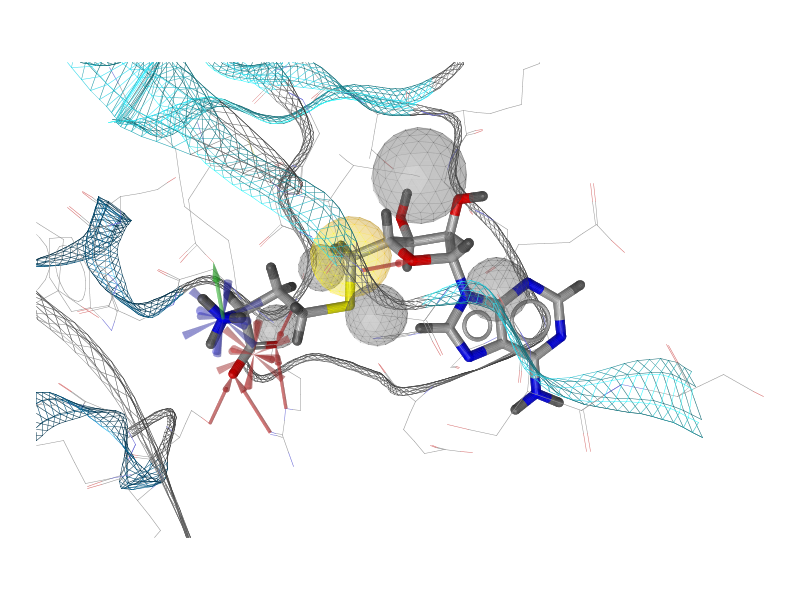
**

**Figure S4.** The predicting binding mode of the full-length of zika NS5 (RC1 at 6 Å), which interact with SAH: (A) 2 D model and (B) 3D model.

**(A)**

**
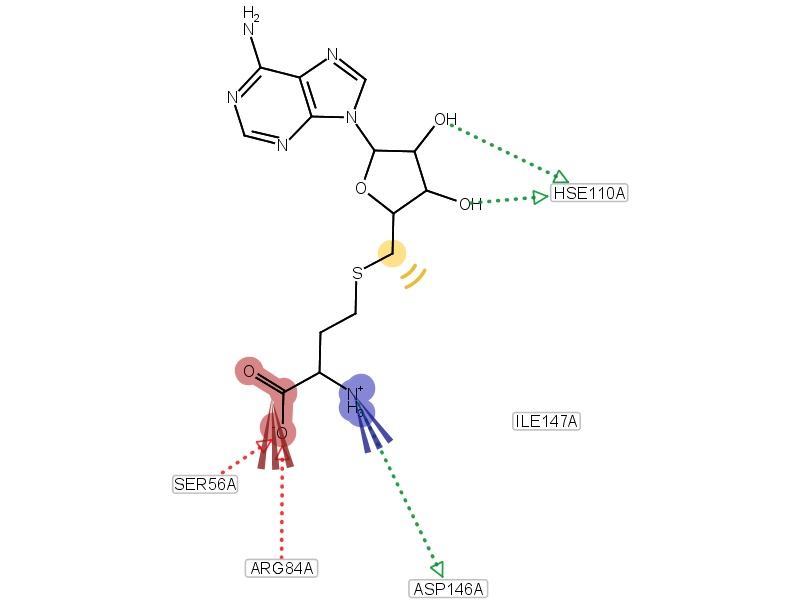
**

**(B)**

**
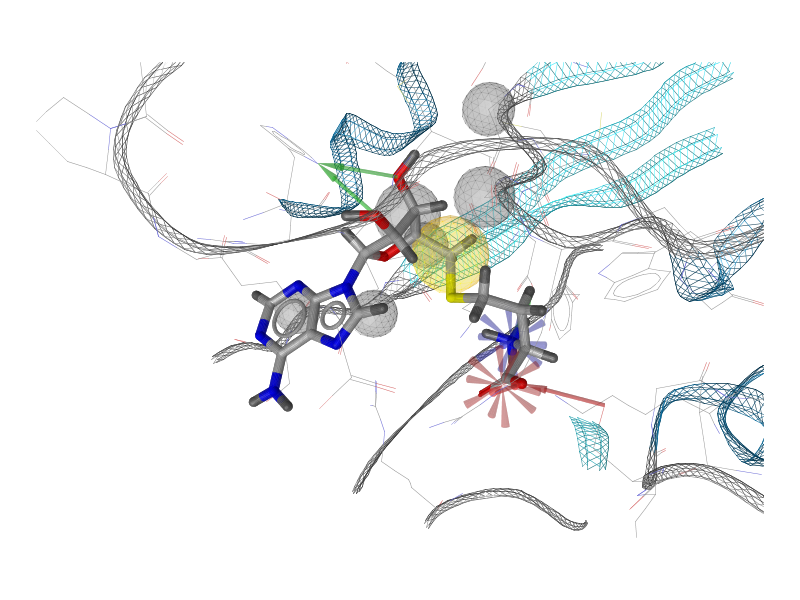
**

**Figure S5.** The predicting binding mode of the full-length of zika NS5 (RC1 at 7 Å), which interact with SAH: (A) 2 D model and (B) 3D model.

**(A)**

**
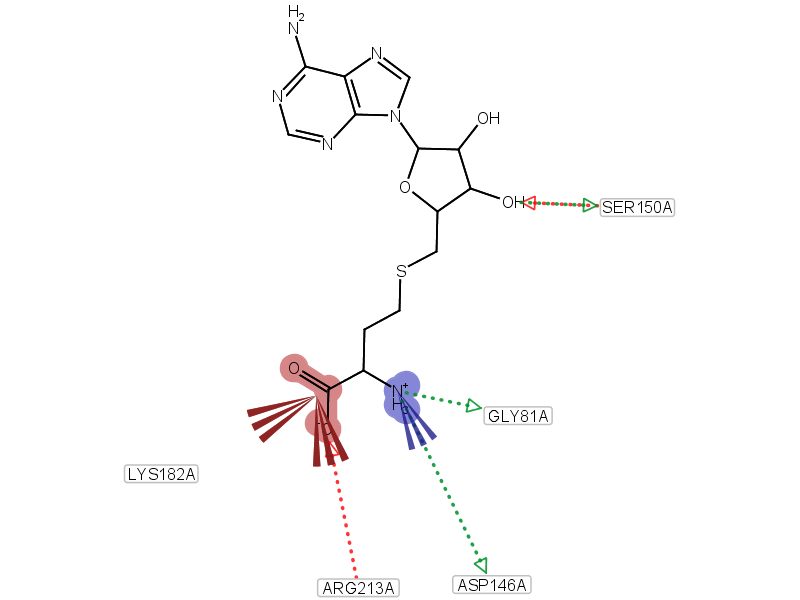
**

**(B)**

**
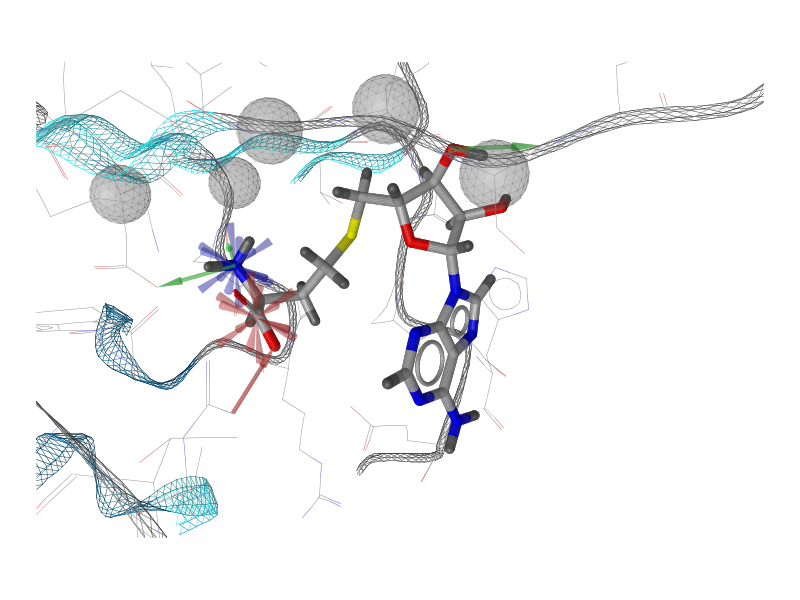
**

**Figure S6.** The predicting binding mode of the full-length of zika NS5 (RC1 at 8 Å), which interact with SAH: (A) 2 D model and (B) 3D model.

**
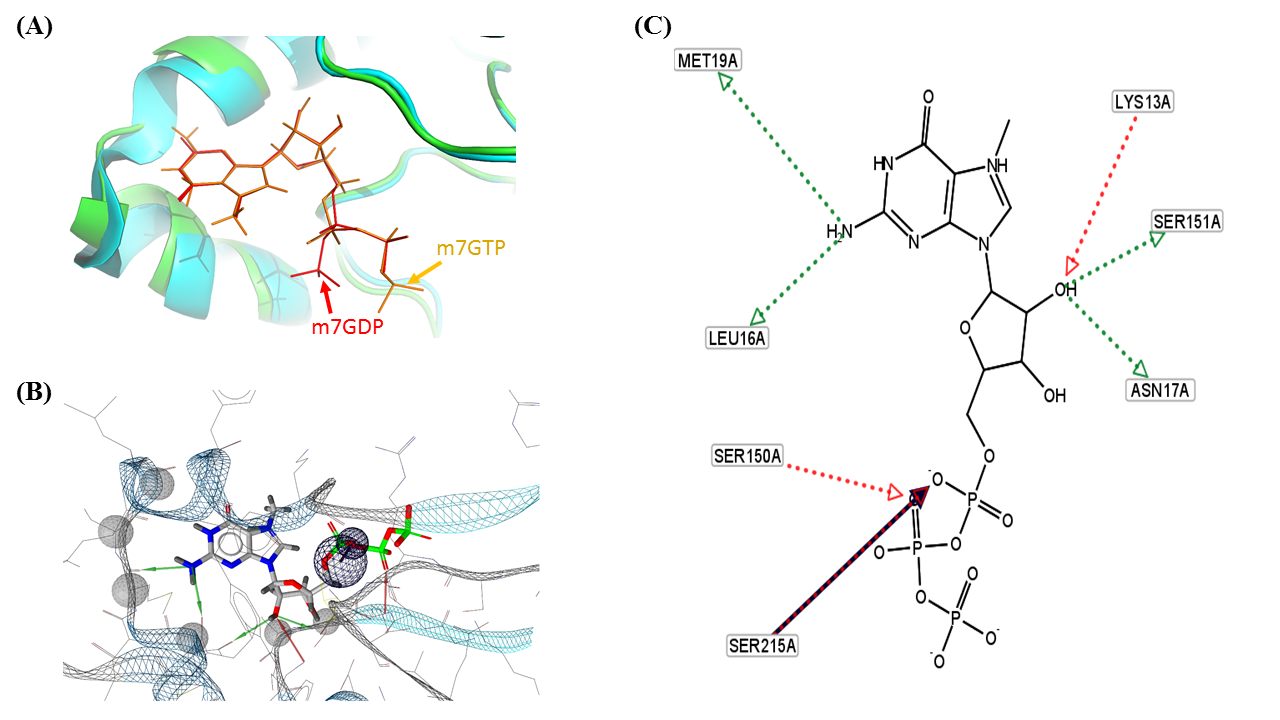
**

**Figure S7.** The experimental binding mode of the full-length of zika NS5 (RC1 at 6 Å), which interact with m7GTP: (A) Structural alignment of the zika NS5 (PDB ID: 5kqs & colored in cyan) with m7GDP and the zika NS5 (PDB ID: 5U0b & colored in green) with m7GTP; (B) the 3D binding modes of the zika NS5 with m7GTP ; (C) the 2D binding modes of the zika NS5 with m7GTP.

**(A)**

**
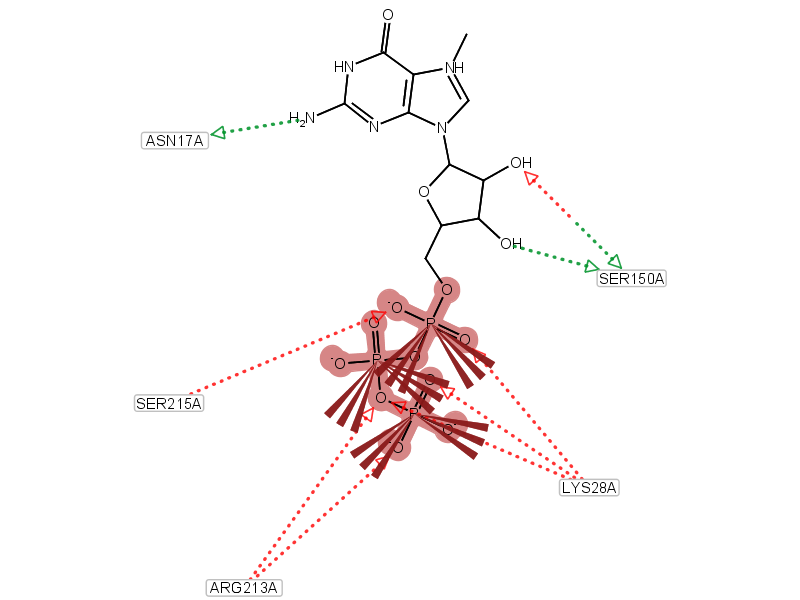
**

**(B)**

**
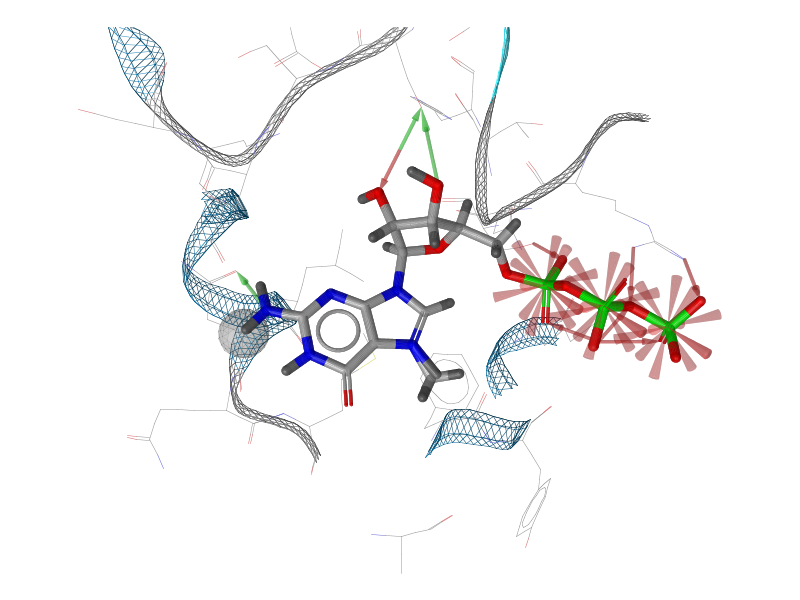
**

**Figure S8.** The predicting binding mode of the full-length of zika NS5 (RC2 at 7 Å), which interact with m7GTP: (A) 2 D model and (B) 3D model.

**(A)**

**
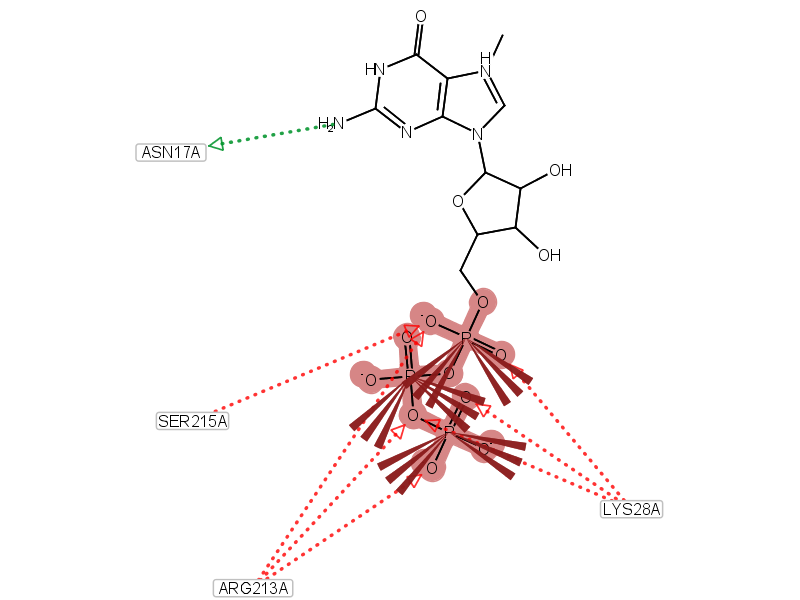
**

**(B)**

**
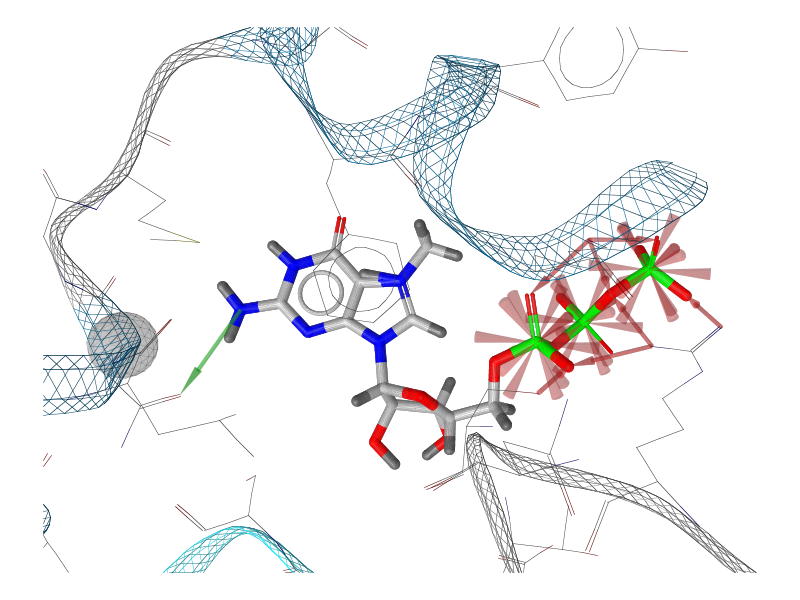
**

**Figure S9.** The predicting binding mode of the full-length of zika NS5 (RC2 at 8 Å), which interact with m7GTP: (A) 2 D model and (B) 3D model.

**(A)**

**
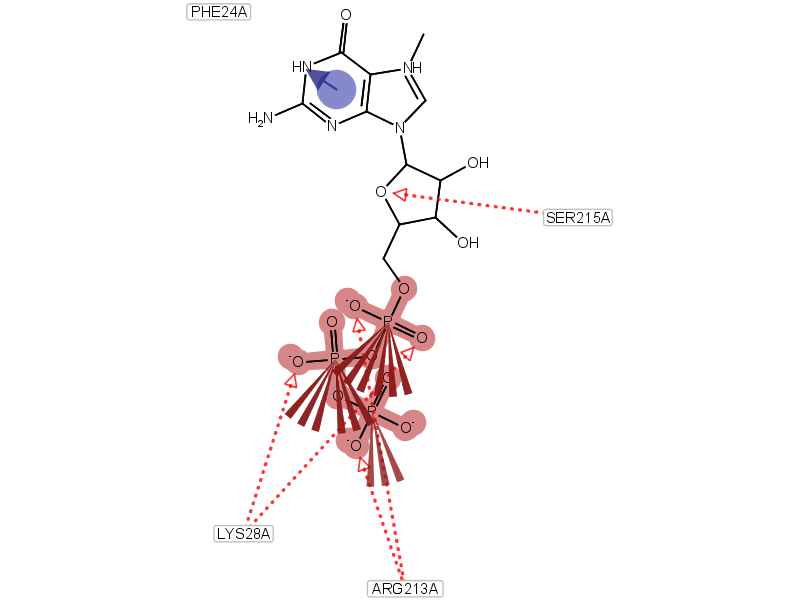
**

**(B)**

**
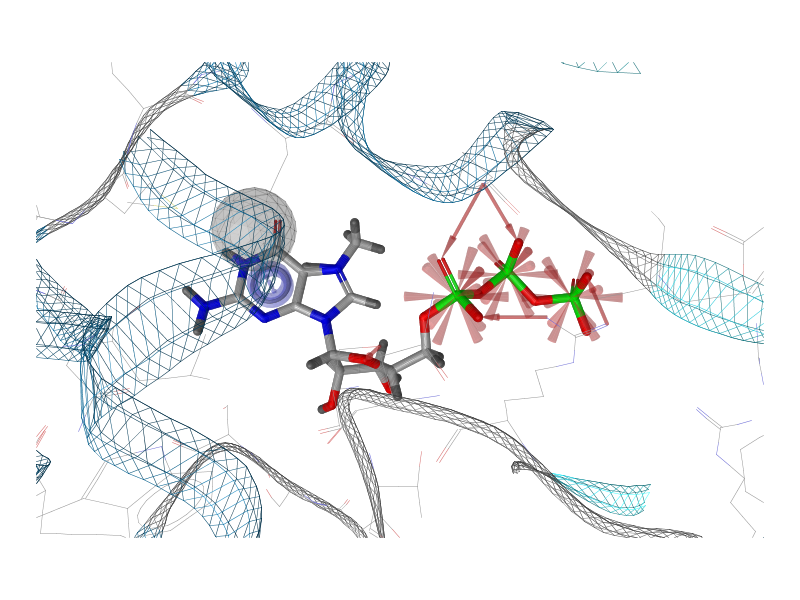
**

**Figure S10.** The predicting binding mode of the full-length of zika NS5 (RC2 at 9 Å), which interact with m7GTP: (A) 2 D model and (B) 3D model.

**(A)**

**
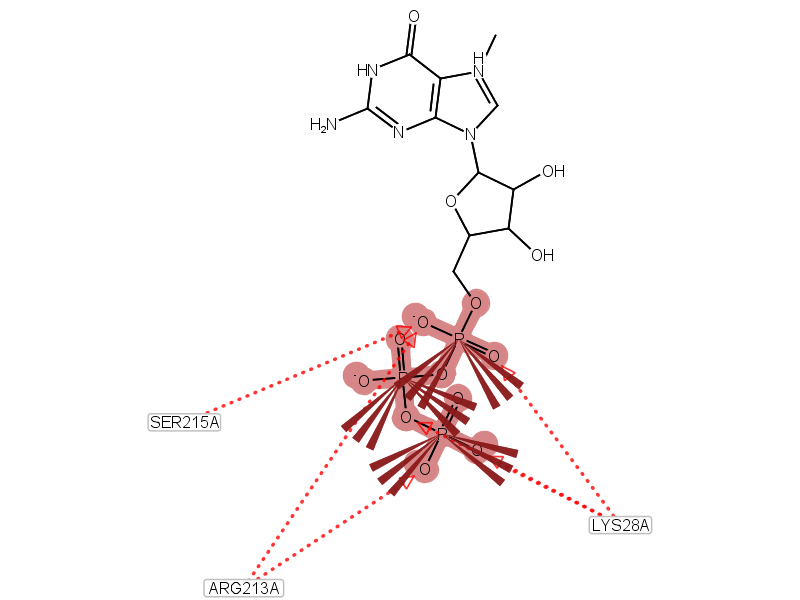
**

**(B)**

**
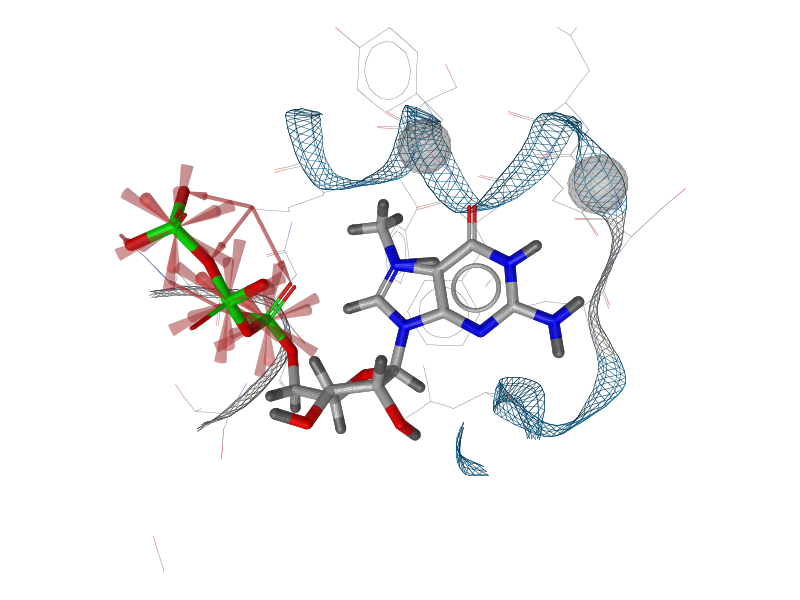
**

**Figure S11.** The predicting binding mode of the full-length of zika NS5 (RC2 at 10 Å), which interact with m7GTP: (A) 2 D model and (B) 3D model.

**(A)**

**
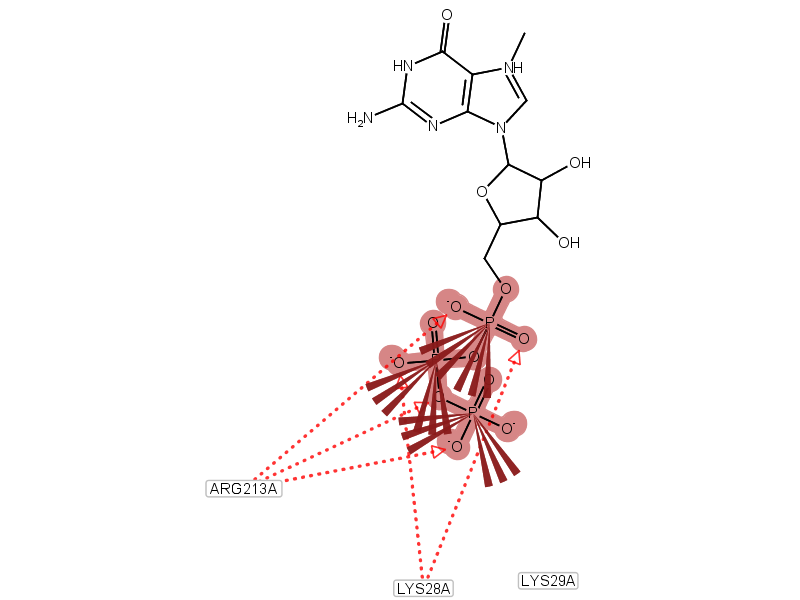
**

**(B)**

**
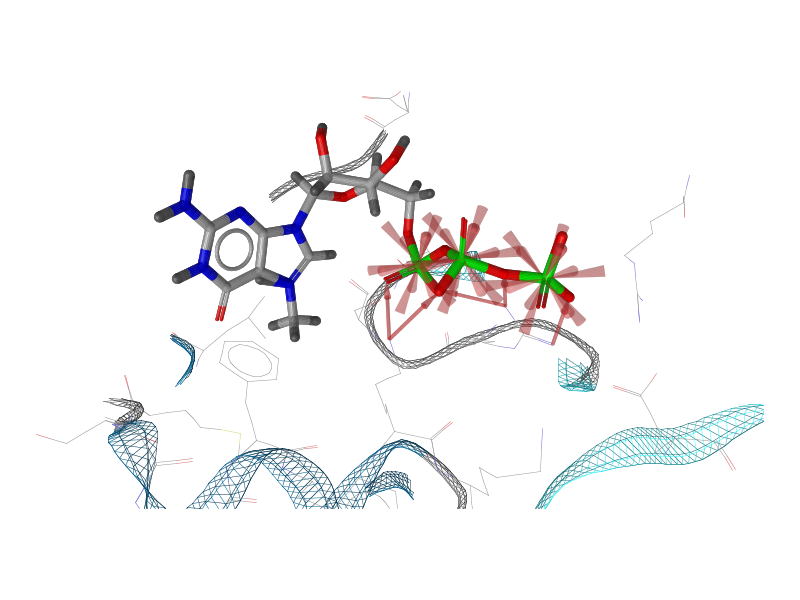
**

**Figure S12.** The predicting binding mode of the full-length of zika NS5 (RC2 at 11 Å), which interact with m7GTP: (A) 2 D model and (B) 3D model.

**
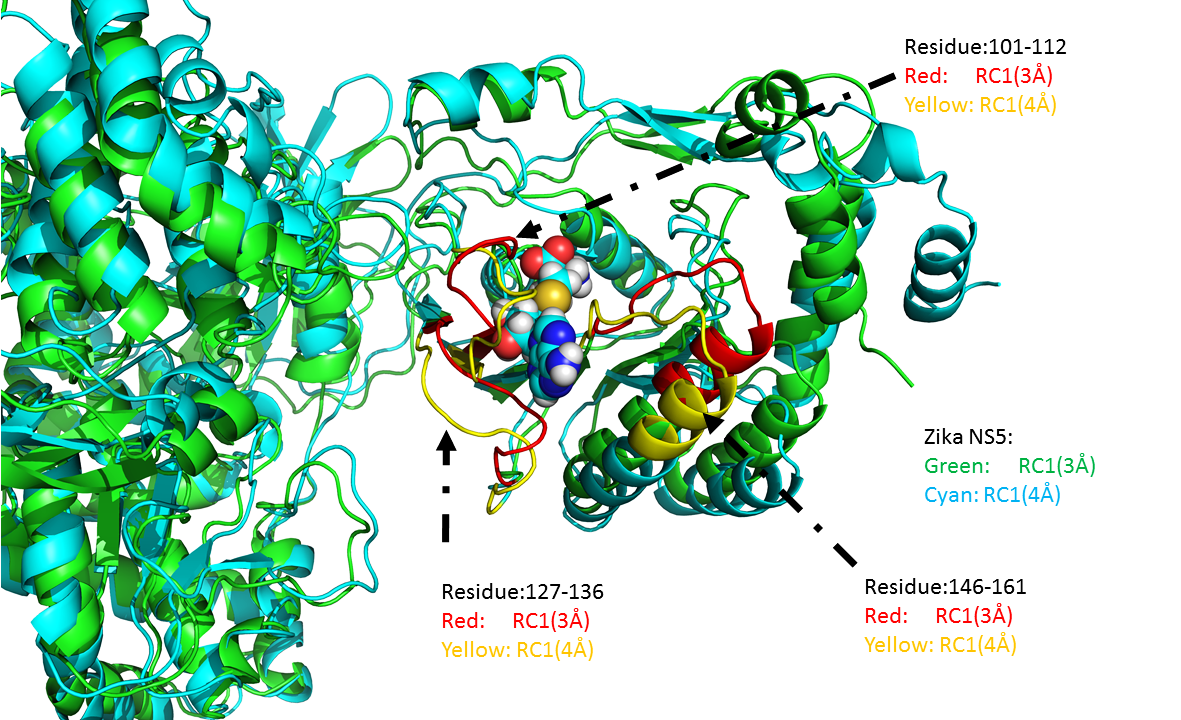
**

**Figure S13.** The pre-residue displacement analysis of the SAH: RC1: 3-4 Å. The SAH molecule is shown as sphere.


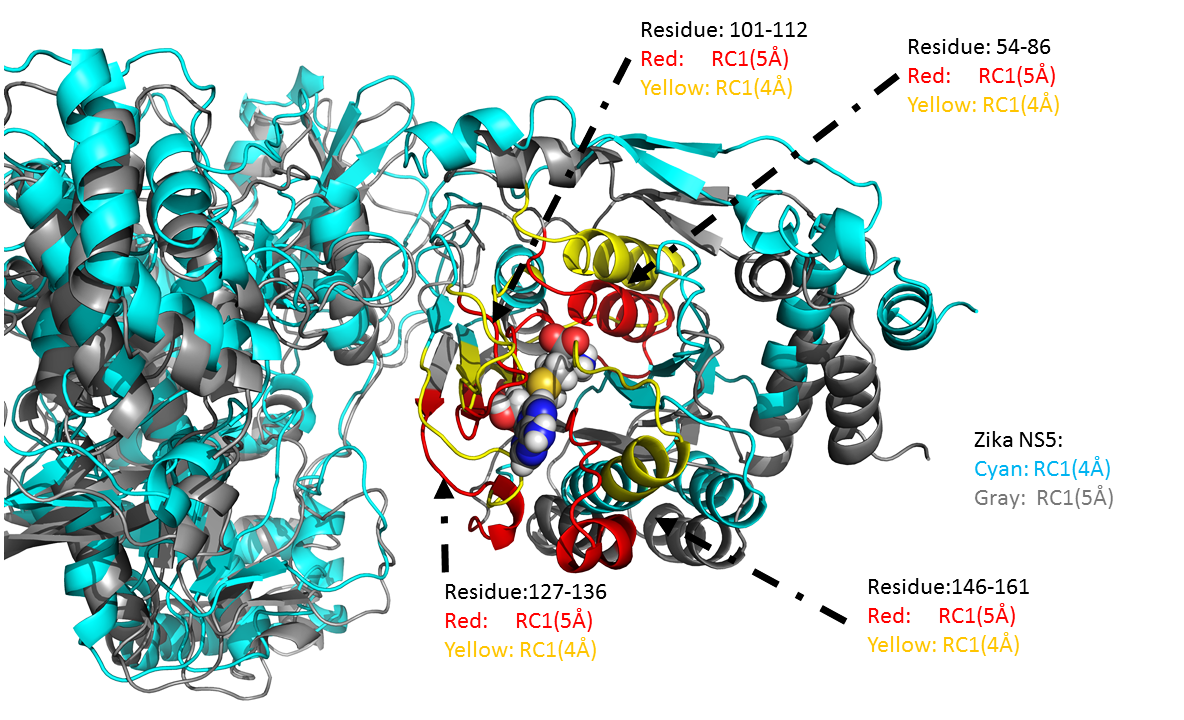


**Figure S14.** The pre-residue displacement analysis of the SAH: RC1: 4-5 Å. The SAH molecule is shown as sphere.

**
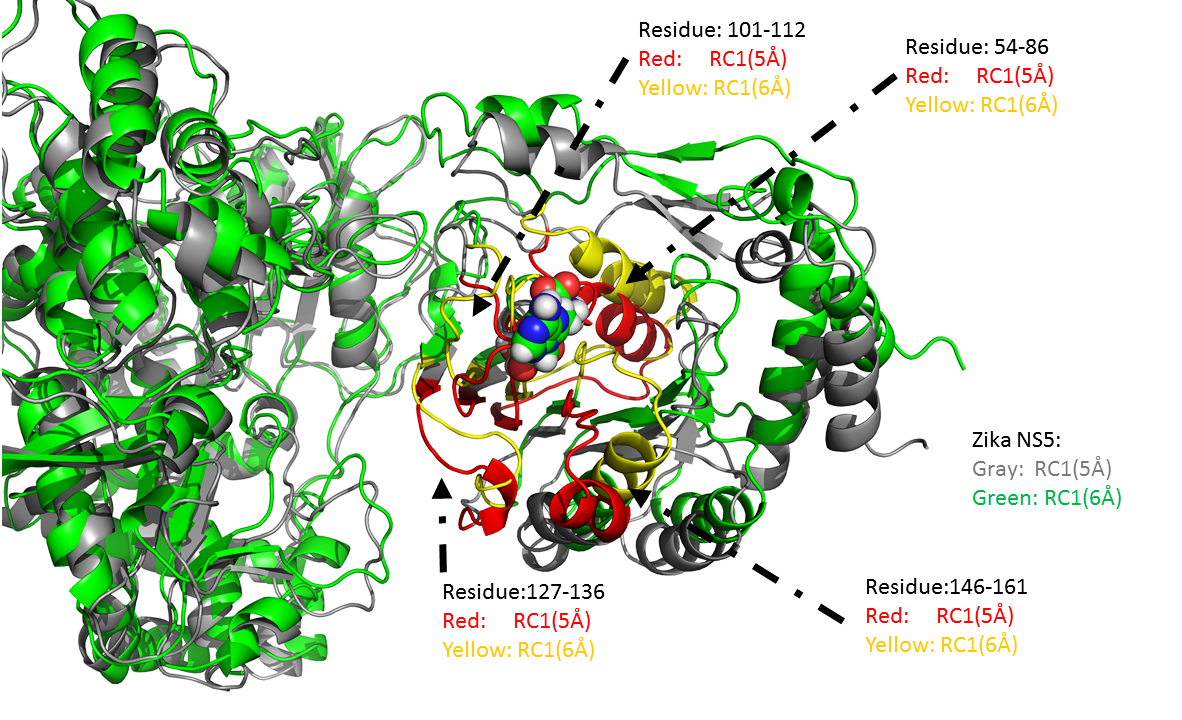
**

**Figure S15.** The pre-residue displacement analysis of the SAH: RC1: 5-6 Å. The SAH molecule is shown as sphere.


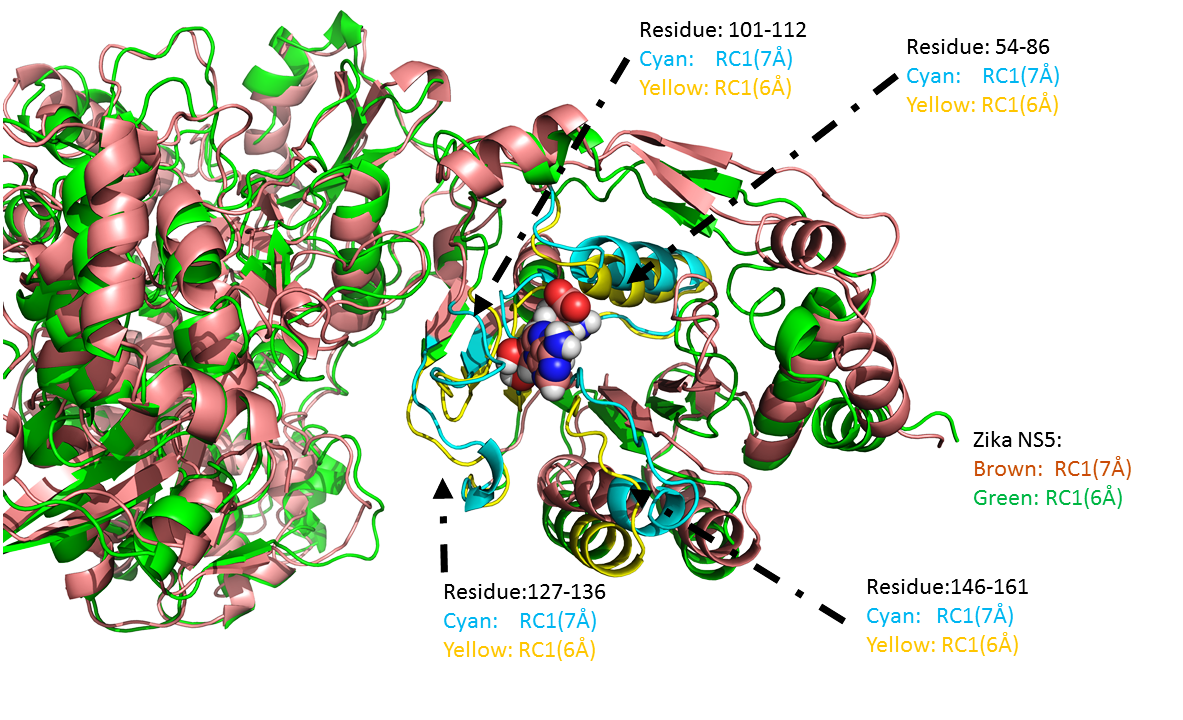


**Figure S16.** The pre-residue displacement analysis of the SAH: RC1: 6-7 Å. The SAH molecule is shown as sphere.

**
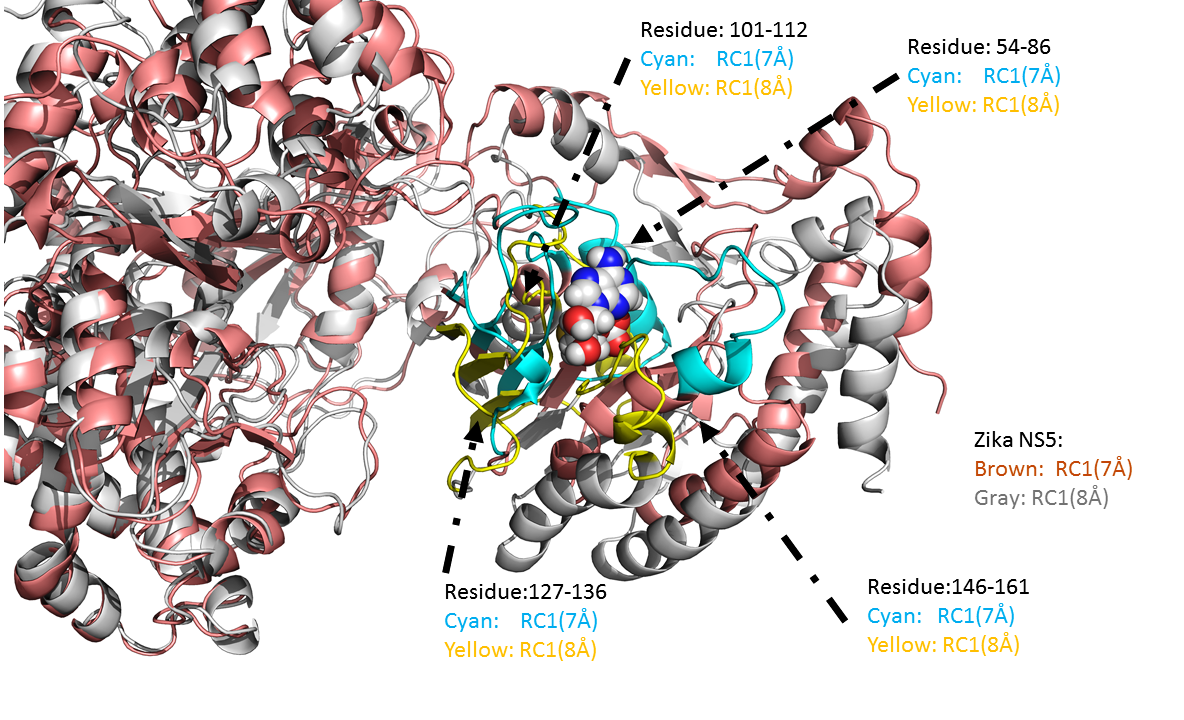
**

**Figure S17.** The pre-residue displacement analysis of the SAH: RC1: 7-8 Å. The SAH molecule is shown as sphere.


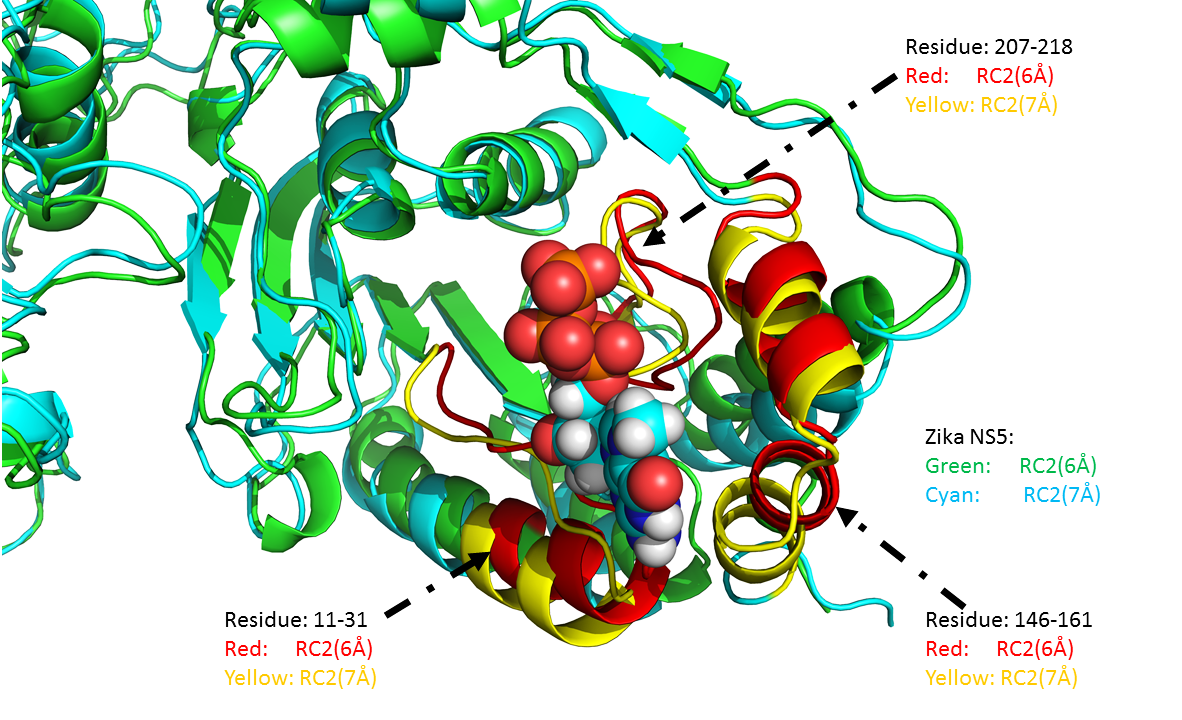


**Figure S18.** The pre-residue displacement analysis of the m7GTP: RC2: 6-7 Å. The m7GTP molecule is shown as sphere.

**
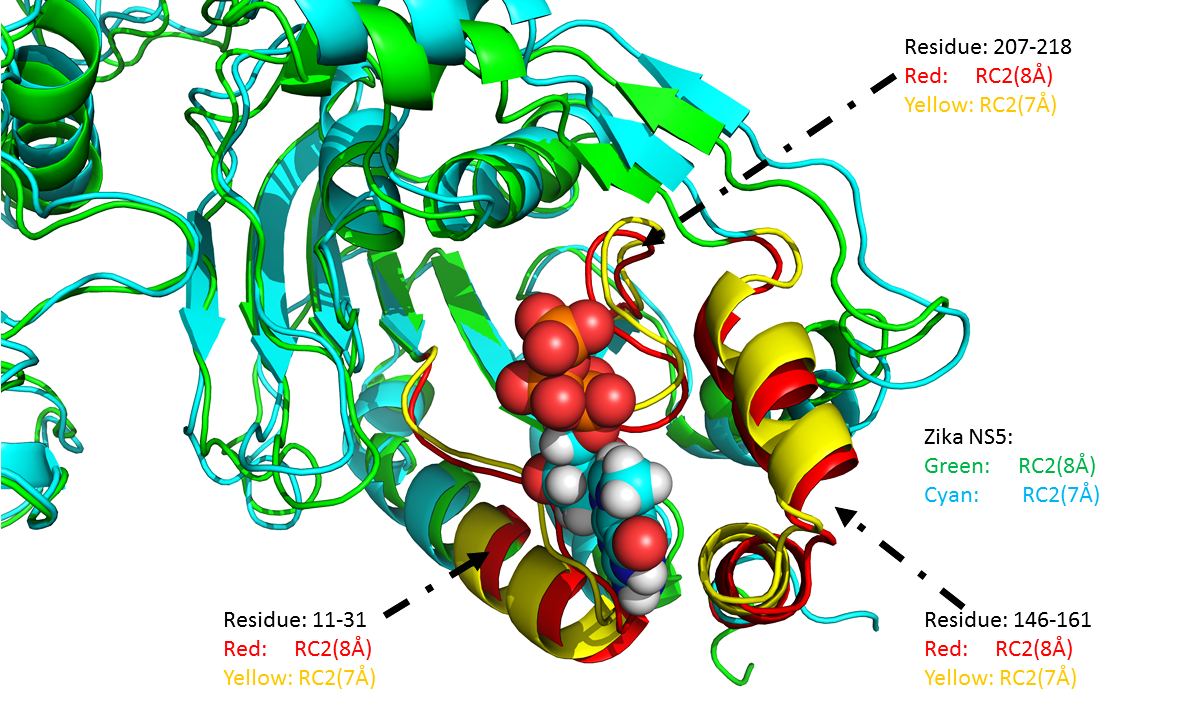
**

**Figure S19.** The pre-residue displacement analysis of the m7GTP: RC2: 7-8 Å. The m7GTP molecule is shown as sphere.


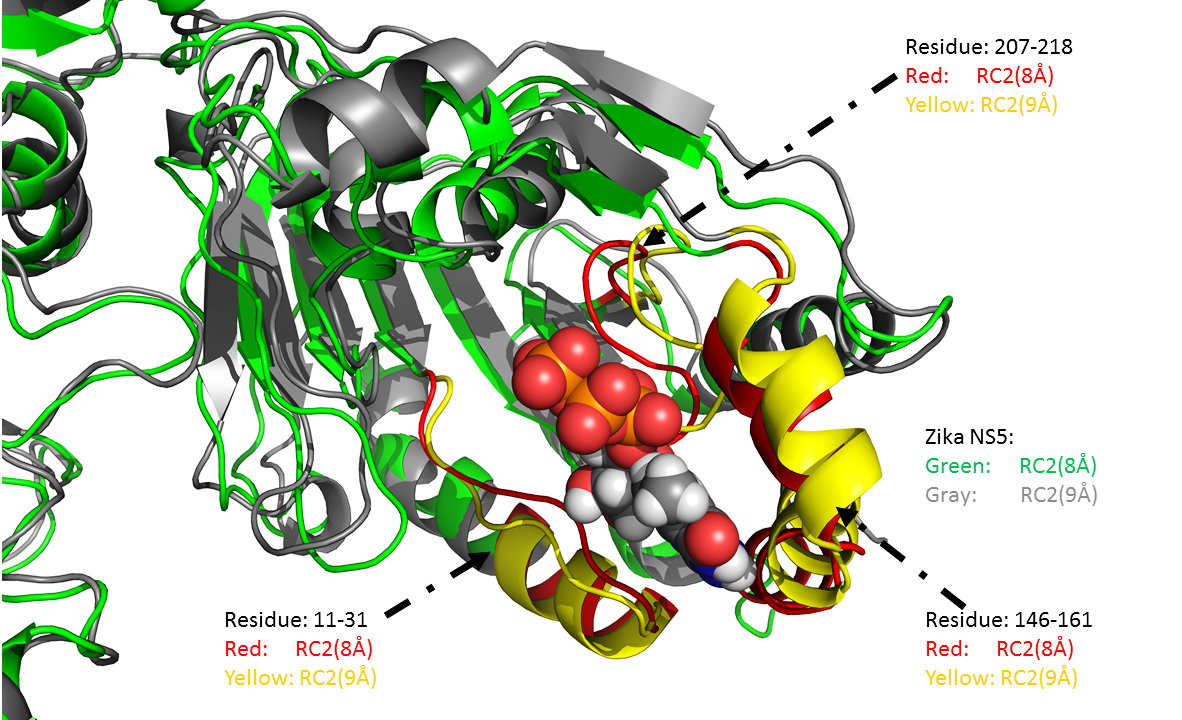


**Figure S20.** The pre-residue displacement analysis of the m7GTP: RC2: 8-9 Å. The m7GTP molecule is shown as sphere.

**
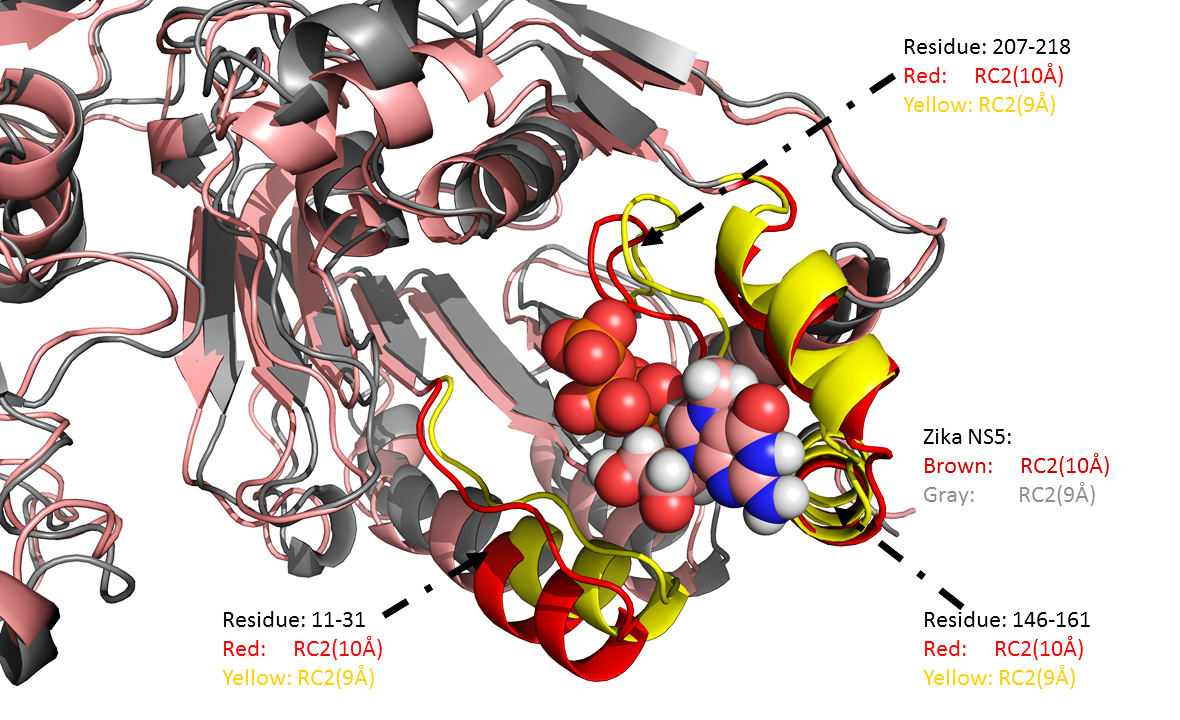
**

**Figure S21.** The pre-residue displacement analysis of the m7GTP: RC2: 9-10 Å. The m7GTP molecule is shown as sphere.

**
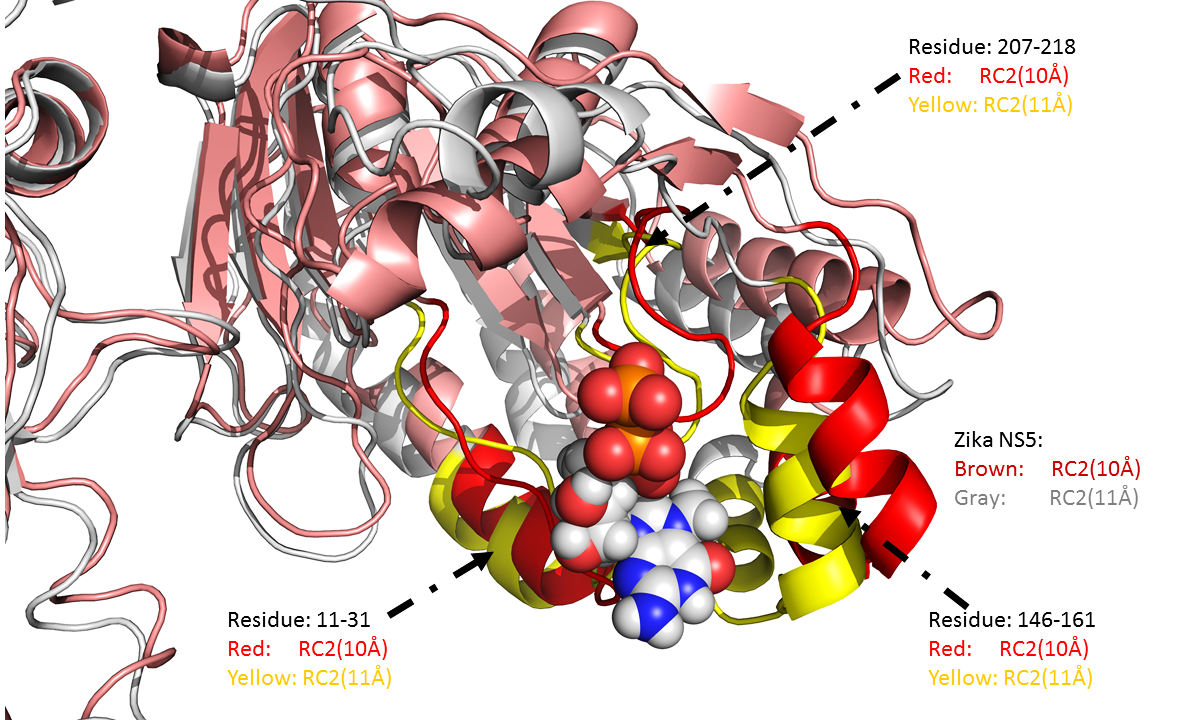
**

**Figure S22.** The pre-residue displacement analysis of the m7GTP: RC2: 10-11 Å. The m7GTP molecule is shown as sphere.


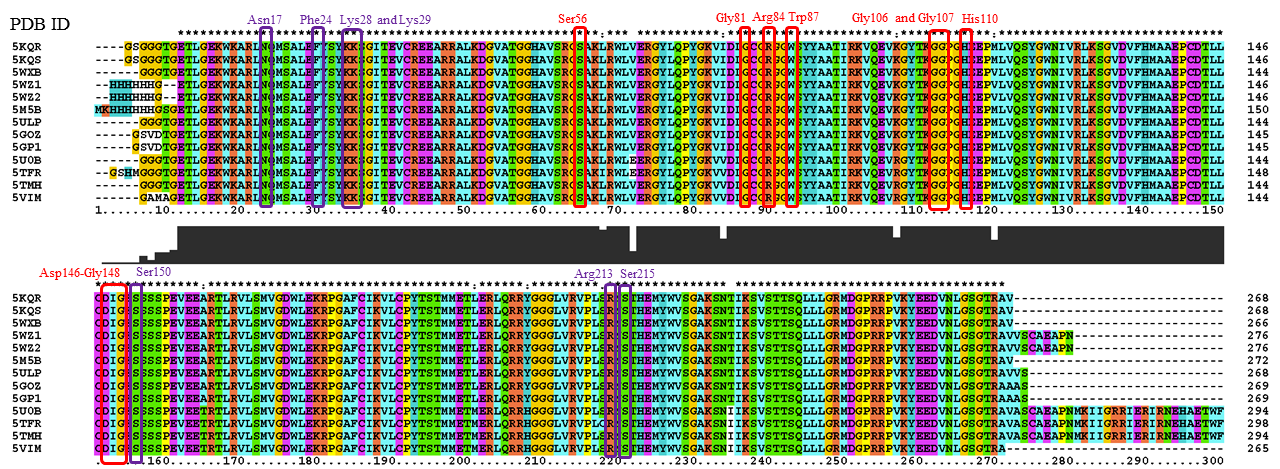


**Figure S23.** The amino sequence alignment the Zika NS5 proteins (PDB ID: 5GOZ, 5GP1, 5KQR, 5KQS, 5M5B, 5TFR, 5TMH, 5U0B, 5ULP, 5VIM, 5WXB, 5WZ1 and 5WZ2). Our predicting residues (Ser56, Gly81, Arg84, Trp87, Thr104, Gly106, Gly107, His110, Asp146, Ile147 and Gly148), which might affect the full-length Zika NS5 protein binding ability of SAH, are colored in red. Our predicting residues (Lys13, Leu16, Asn17, Met19, Ser150, Ser151 and Ser2152), which might affect the full-length Zika NS5 protein binding ability of m7GTP, are colored in purple.


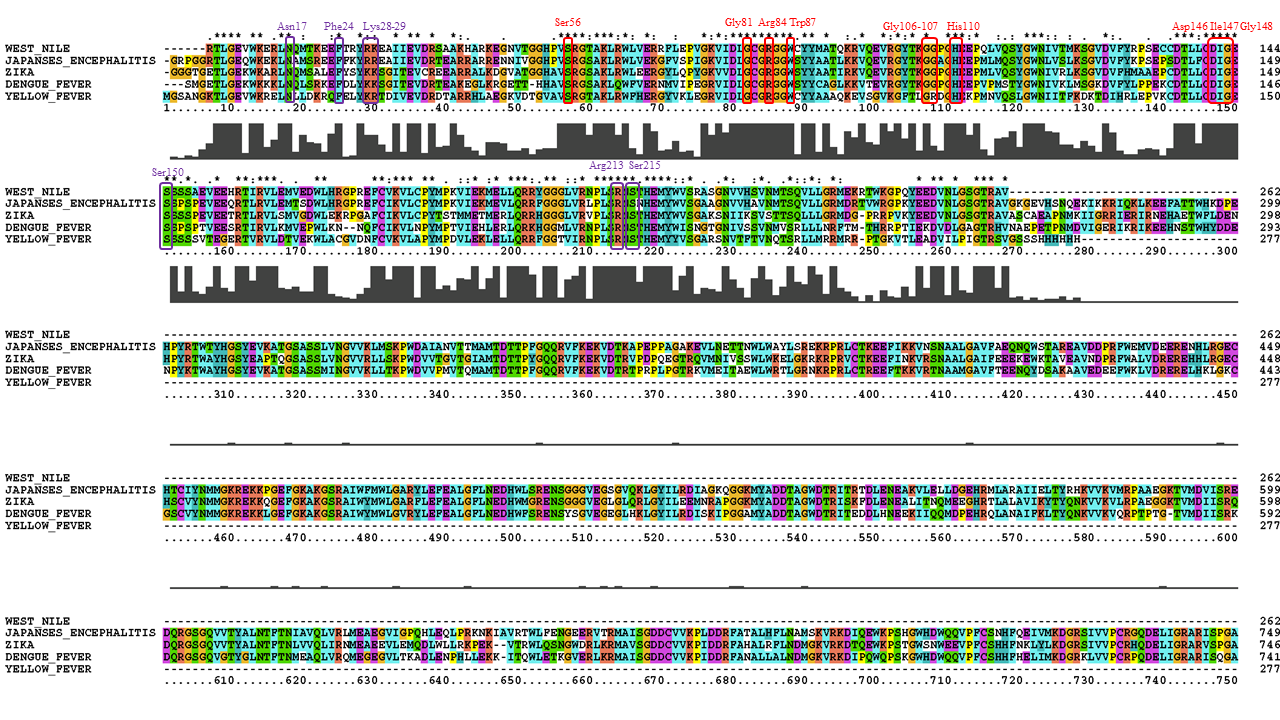


**Figure S24.** The amino sequence alignment flavivirus NS5 proteins (PDB ID: 3EVF(Yellow fever), 4V0R(Dengue fever), 2HKS(West Nile) and 4K6M(Japanese encephalitis)). Our predicting residues (Ser56, Gly81, Arg84, Trp87, Thr104, Gly106, Gly107, His110, Asp146, Ile147 and Gly148), which might affect the full-length Zika NS5 protein binding ability of SAH, are colored in red. Our predicting residues (Lys13, Leu16, Asn17, Met19, Ser150, Ser151 and Ser2152), which might affect the full-length Zika NS5 protein binding ability of m7GTP, are colored in purple.

1. (B)

**Figure S25.** 2D Structures: (A) SAH (S-adenosyl-L-homocysteine) and (B) M7GTP (7-methylguanosine 5’-triphosphate).
